# Leishmanicidal Potential of Hardwickiic Acid Isolated From *Croton sylvaticus*

**DOI:** 10.3389/fphar.2020.00753

**Published:** 2020-05-25

**Authors:** Justice Afrifa Crentsil, Lauve Rachel Tchokouaha Yamthe, Barbara Zenabu Anibea, Emmanuel Broni, Samuel Kojo Kwofie, John Kweku Amissah Tetteh, Dorcas Osei-Safo

**Affiliations:** ^1^Department of Chemistry, School of Physical and Mathematical Sciences, College of Basic and Applied Sciences (CBAS), University of Ghana, Accra, Ghana; ^2^Institute for Medical Research and Medicinal Plants Studies, Yaoundé, Cameroon; ^3^Department of Parasitology, College of Health Sciences, Noguchi Memorial Institute for Medical Research, University of Ghana, Accra, Ghana; ^4^Antimicrobial and Biocontrol Agents Unit, Laboratory for Phytobiochemistry and Medicinal Plants Studies, Faculty of Science, University of Yaoundé I, Yaoundé, Cameroon; ^5^Department of Biomedical Engineering, School of Engineering Sciences, CBAS, University of Ghana, Accra, Ghana; ^6^West African Centre for Cell Biology of Infectious Pathogens, Department of Biochemistry, Cell and Molecular Biology, CBAS, University of Ghana, Accra, Ghana; ^7^Department of Medicine, Loyola University Medical Center, Maywood, IL, United States; ^8^Department of Physics and Engineering Science, Coastal Carolina University, Conway, SC, United States; ^9^Department of Immunology, College of Health Sciences, Noguchi Memorial Institute for Medical Research, University of Ghana, Accra, Ghana

**Keywords:** *Croton sylvaticus*, hardwickiic acid, leishmaniasis, structural modeling, molecular docking, trypanothione reductase, pteridine reductase 1, glutamate cysteine ligase

## Abstract

*Leishmania* is a parasitic protozoon responsible for the neglected tropical disease Leishmaniasis. Approximately, 350 million people are susceptible and close to 70,000 death cases globally are reported annually. The lack of effective leishmanicides, the emergence of drug resistance and toxicity concerns necessitate the pursuit for effective antileishmanial drugs. Natural compounds serve as reservoirs for discovering new drugs due to their chemical diversity. Hardwickiic acid (HA) isolated from the stembark of *Croton sylvaticus* was evaluated for its leishmanicidal potential against *Leishmania donovani* and *L. major* promastigotes. The susceptibility of the promastigotes to HA was determined using the 3-[4,5-dimethylthiazol-2-yl]-2,5-diphenyltetrazolium bromide/phenazine methosulfate colorimetric assay with Amphotericin B serving as positive control. HA showed a significant antileishmanial activity on *L. donovani* promastigotes with an IC_50_ value of 31.57± 0.06 µM with respect to the control drug, amphotericin B with IC_50_ of 3.35 ± 0.14 µM). The cytotoxic activity was observed to be CC_50_ = 247.83 ± 6.32 µM against 29.99 ± 2.82 µM for curcumin, the control, resulting in a selectivity index of SI = 7.85. Molecular modeling, docking and dynamics simulations of selected drug targets corroborated the observed antileishmanial activity of HA. Novel insights into the mechanisms of binding were obtained for trypanothione reductase (TR), pteridine reductase 1 (PTR1), and glutamate cysteine ligase (GCL). The binding affinity of HA to the drug targets *Lm*GCL, *Lm*PTR1, *Ld*TR, *Lm*TR, *Ld*GCL, and *Ld*PTR1 were obtained as -8.0, -7.8, -7.6, -7.5, -7.4 and -7.1 kcal/mol, respectively. The role of Lys16, Ser111, and Arg17 as critical residues required for binding to *Ld*PTR1 was reinforced. HA was predicted as a Caspase-3 stimulant and Caspase-8 stimulant, implying a possible role in apoptosis, which was shown experimentally that HA induced parasite death by loss of membrane integrity. HA was also predicted as antileishmanial molecule corroborating the experimental activity. Therefore, HA is a promising antileishmanial molecule worthy of further development as a biotherapeutic agent.

**Graphical Abstract:**
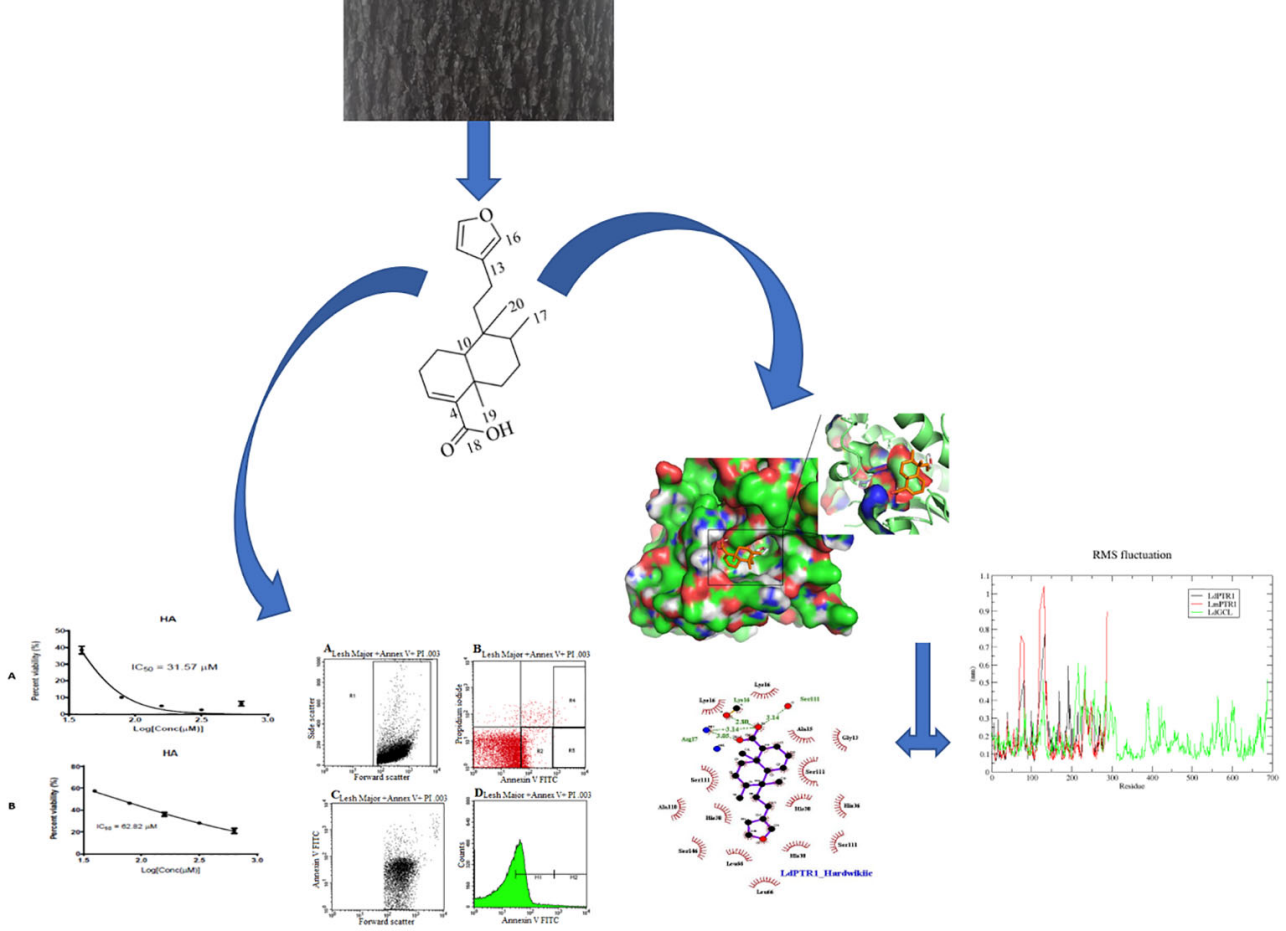
Methodology schema for the evaluation of the leishmanicidal potential of hardwickiic acid.

## Introduction

Leishmaniasis which is caused by *Leishmania* is one of the most neglected tropical diseases needing urgent attention (Feasey et al., 2010; de Vries et al., 2015). It is endemic in about 90 countries with 350 million people at risk of infection and has an annual mortality rate of 70,000 cases globally (Alvar et al., 2012; de Vries et al., 2015; Georgiadou et al., 2016). According to the World Health Organization (WHO), there are about 700,000 to close to a million new cases of both cutaneous leishmaniasis (CL) and visceral leishmaniasis (VL) annually (WHO, 2019). Leishmaniasis is spread to humans after being bitten by infected phlebotomine sand flies (Kamhawi, 2006; Bates, 2007; Corrales et al., 2010; Dostálová and Volf, 2012; Ready, 2013). Generally, leishmaniases are endemic among the under-developed geographic regions and the disease is recognized by the development of single or multiple lesions (WHO, 2018).

The lack of effective and affordable therapeutics is hindering the eradication of leishmaniasis. Currently, therapeutic interventions for controlling leishmaniasis include amphotericin B and its liposomal formulation known as AmBisome, as well as pentavalent antimonial, miltefosine, pentamidine and paromomycin. Also, combination therapy of existing drugs is a preferred treatment option. However, these drugs have been reported to pose serious toxicity challenges and the emergence of drug-resistant strains has become prevalent (Tiwari et al., 2017; Tiwari and Dubey, 2018). The inherent differences in drug sensitivity shown by the different species could be an explanation to the resistance phenomenon (Mandal et al., 2015). Till date, there is no suitable vaccine to entirely eliminate leishmaniasis (Ghorbani and Farhoudi, 2018).

The lack of effective drugs, the emergence of drug resistance, drug toxicity concerns and absence of cost-effectiveness necessitate the search for effective therapeutic molecules and new drug targets (Chawla and Madhubala, 2010). Various efforts have been geared towards finding cures to diseases of parasitic origin (Crowther et al., 2010). The dissimilarities in human and homology amongst parasites have affected the prioritization of drug targets and drug candidates due to reduced interference of these drugs with host systems (Lamotte et al., 2017). Metabolic pathways of the various *Leishmania* species have been studied in the past decades to elucidate potential targets. Trypanothione reductase (TR), pteridine reductase 1 (PTR1), and glutamate cysteine ligase (GCL) have been identified as plausible targets.

TR is essential for the survival of the parasites in hosts since it is responsible for converting trypanothione disulfide T[S]_2_ to its dithiol T[SH]_2_ moiety (Saccoliti et al., 2017), which is required by the tryparedoxin-tryparedoxin peroxidase system for the neutralization of hydrogen peroxide generated during infection by the macrophages of the host (Colotti et al., 2013; Turcano et al., 2018). TR-knockout mutants engineered through gene disruption in *L. donovani* and *L. major* strains decreases infectivity thereby stagnating the intracellular survival of the parasites in macrophages (Dumas et al., 1997; Tovar et al., 1998). TR is an attractive drug target for selective inhibition since it plays essential role in immunology mechanisms and pathogenesis.

The growth of the parasite as well as that of trypanosomatid protozoa depends on reduced pteridines especially pterins and folates. PTR1 which is an NADPH-dependent short-chain reductase present in parasitic trypanosomatid protozoans takes part in salvaging pterins (Schüttelkopf et al., 2005). Amplification of PTR1 is critical to the resistance of the parasite to antifolates (Nare et al., 1997b). PTR1 acts as a metabolic bypass for drugs targeting dihydrofolate reductase (DHFR) (Kumar et al., 2008). Since PTR1 has been shown to be less sensitive to antifolates which primarily target DHFR (Nare et al., 1997a), any treatment with antifolate in *Leishmania* should also target PTR1.

GCL is involved in the biosynthesis of trypanothione (TSH) (Mukherjee et al., 2009; Kyriazis et al., 2016). TSH enables the parasite to avoid the deleterious effects of nitric oxide (NO) and reactive oxygen species (ROS), which are generated by the macrophages as part of the host's defense against leishmaniasis (Mukherjee et al., 2009; Kyriazis et al., 2016). An amplification or overexpression of this enzyme has also been shown to increase *in vitro* resistance of *Leishmania* to antimonials (Grondin et al., 1997).

Constituents of plants affords limitless opportunities for discovering novel drugs due to the unparalleled diversity of chemical libraries (Carlson, 2010; Lahlou, 2013). Traditional medicine serves as a remedy for over 80% of the global population (Sasidharan et al., 2011). The constituents of plant extracts are natural products for treating plethora of diseases. The phytochemical screening of the constituents serve as the precursor for new therapeutics needed to treat disease (Sasidharan et al., 2011).

The *Crotons* (Euphorbiaceae) represent around 1300 species of trees, shrubs, and herbs widely distributed in the tropics (Salatino et al., 2007; Xu et al., 2018). They have a rich history of ethnomedicinal uses including malaria, inflammation, tuberculosis, and stomach upset (Salatino et al., 2007). Extensive phytochemical investigations have identified multiple classes of secondary metabolites, predominantly terpenoids and sterols, with diverse pharmacological applications such as antimicrobial, cytotoxicity, anti-inflammatory, antioxidant, antinociceptive, molluscicidal, and wound healing (Jassbi, 2006). Hardwickiic acid (HA) has been isolated from many *Croton* species including *C. sonderianus*, *C. aromaticus*, and *C. oblongifolius* (Chaichantipyuth et al., 2004). HA also exhibits antimicrobial and insecticidal activities (Bandara et al., 1987; McChesney et al., 1991).

Due to the enormous structural and biological diversity of the chemical constituents, the genus continues to attract remarkable attention (Shi et al., 2008). However, one of the research gaps in existing literature on the *Crotons* is a lack of correlation between bioactive molecules and their mechanisms of action. In this paper, we present the leishmanicidal potential of hardwickiic acid (HA) isolated from the stembark of *Croton sylvaticus* by evaluating its biological activity. Also, molecular modeling and docking studies were performed on trypanothione reductase (TR), pteridine reductase 1 (PTR1), and glutamate cysteine ligase (GCL) of both *L. donovani* and *L. major* in order to elucidate the binding mechanisms between HA and drug targets. This study also sought to determine novel critical active site residues of *Ld*TR, *Lm*TR, *Ld*GCL, and *Lm*GCL involved in HA binding. More so, sought to predict potential mechanisms of action of HA by using an Open Bayesian-based approach.

## Materials and Methods

### Plant Material and Isolation

A semi-purified fraction of a previously petroleum ether-extracted stembark of *Croton sylvaticus* (Mwangi et al., 1998) was analyzed in this work. The plant material was collected at Mombasa in 1989 and identified by Mr. G. Mungai of the East African Herbarium based in Nairobi, Kenya. Voucher specimens were deposited at the Faculty of Pharmacy in the University of Nairobi in Kenya.

The Thin-layer Chromatographic (TLC) profile of the extract, developed in petrol-EtOAc (3:7), indicated one major spot and three others. In order to separate the constituents, 0.535 g of the dried light brown powdery fraction was column chromatographed on silica gel, eluting with petrol and petrol-EtOAc mixtures. A total of fifty-seven 30 ml eluents were collected and pooled into nine fractions F1–F9 based on their TLC profiles. On drying under vacuum, solids precipitated from fractions F1 (235 mg), F2 (0.9 mg), and F6 (0.5 mg). Due to paucity of material, further characterization was conducted on F1 only to afford hardwickiic acid (HA). Analytical liquid chromatography–mass spectrometry (LCMS) analysis was undertaken using an Agilent equipped with Kinetex Core C18, 2.6 µm, 3 x 50 mm, 100 Å maintained at 40 °C and DAD UV-detector (220, 254 and 300 nm). The mobile phase was 10 mM NH_4_OAc in 90% MeOH in H_2_O. The ionization technique was Jet Stream-Electrospray in positive mode. Nuclear Magnetic Resonance (NMR) spectra were obtained at 500 MHz on a Brüker Ascend™ 500 Spectrometer in CDCl_3_ with TMS as the internal standard. Optical rotation was measured on a PerkinElmer141 polarimeter which is equipped with a Na lamp of λ = 589 nm.

### Antileishmanial Assay

#### Parasite Culture

*L. donovani* (1S MHOM/SD/62/1S strain) and *L. major* (IFLA/BR/67/PH8 strain) procured from the BEI Resources, National Institute of Allergy and Infectious Diseases (NIAID), National Institutes of Health (NIH). They were cultured in ME199 medium, pH 7.4, supplemented with 10% heat-inactivated fetal bovine serum (FBS) and 1% penicillin-streptomycin and kept in an air atmosphere at 28°C in 75 cm^2^ Roux flasks.

#### Cell Culture

RAW 264.7 cells were procured from the RIKEN BioResource Centre Cell Bank in Japan. They were maintained in DMEM supplemented with 10% FBS and 1% penicillin-streptomycin under 5% CO_2_ and humidified atmosphere at 37°C.

### Cytotoxicity Activity

*In vitro* evaluation of the cytotoxicity activity was carried out on RAW 264.7 cell line using the resazurin assay, which measures cellular metabolic activity (Süzgeaç-Selaçuk et al., 2011). A sub-confluent cell culture in 75 cm^2^ culture flask, was trypsinized, and cells were counted and suspended in DMEM supplemented with 10% FBS and 1% penicillin-streptomycin. Cells were seeded into a 96-well plate (100 μl per well) at concentrations of 1x10^5^ cells per ml and incubated overnight with 5% CO_2_ at 37°C, to allow cells to attach to the surface of the plate. The cells were then treated in triplicate with increasing concentration of each compound (1.01 to 632.71 μM). After 48 h of incubation, 10 μl of 2.5 mM resazurin solution were added to each well and further incubated for 4 h at 37°C. Fluorescence was measured using the microplate reader (TECAN Infinite M200 Pro Plate Reader, Austria) at excitation and emission wavelengths of 530 nm and 590 nm, respectively. Medium seeded with cells without treatment served as negative control. Curcumin was used as positive control, while medium without cells served as blank. The experiments were done in triplicate and all the data were shown as means ± SD. The percent growth inhibition was computed from the absorbances relative to the negative control, and the concentration of compound that inhibited 50% cell (CC_50_ values) was calculated with GraphPad Prism 7.0 (GraphPad Prism software Inc. San Diego, CA).

### Antipromastigote Assay

The susceptibility of promastigotes to HA was determined using the 3-[4,5-dimethylthiazol-2-yl]-2,5-diphenyltetrazolium bromide/phenazine methosulfate (MTS/PMS, Promega) colorimetric assay (Wong et al., 2014). Glucose-6-phosphate dehydrogenase which takes part in the pentose phosphate pathway of *Leishmania* parasites are needed for the reduction of MTS to formazan. Briefly, stationary-phase promastigotes were seeded into 96-well flat-bottomed microtiter plates at 2x10^7^ parasites per well and incubated at 28°C in the absence or presence of different concentrations of HA (39.54 to 632.71 μM). After 72 h of incubation, 10 μl of MTS/PMS solution were added to each well of the microtiter plate. The plates were further incubated at 28°C for colour development. After 4 h of incubation, the optical densities were read at 490 nm using an automated microtiter plate reader (TECAN Infinite M200 Pro Plate Reader, Austria). The negative control consisted of medium with untreated parasites, while medium without parasites served as blank. Amphotericin B was used as positive control. All the experiments were done in triplicate and the data were shown as means ± SD. The percent growth inhibition was computed from the optical densities relative to the negative control, and the concentration of compound that inhibited the parasite growth by 50% (IC_50_ values) were calculated with GraphPad Prism 7.0.

### Determination of Phosphatidylserine Externalization

Externalization of the Phosphatidylserine was done using Annexin–V FLUOS staining kit (Roche), whilst Fluorescein-conjugated Annexin-V was used to detect the externalized phosphatidylserine due to very high binding affinity to the phospholipid portion. Additionally, annexin-V FLUOS distinguishes between surviving, apoptotic and necrotic cells. The stationary phase promastigotes (1×10^7^ cells/ml) were either treated or untreated with HA and amphotericin B at IC_50_ for 72 h at 28°C. The downstream processing complied with the instructions of the manufacturer. Parasites were washed in cold phosphate-buffered saline (PBS) by centrifuging at 200 g for 5 min and the pellet was incubated with Annexin-V-FLOUS and PI (20 μl each) togther with sample buffer (100 μl) for 15 min at room temperature in the dark. After incubation, the samples were acquired on a flow cytometer (BD LSRFortessa II-x20), FlowJo software program was used for analysis and the percent of positive parasites was calculated per sample.

### Preparation of the Structures of Pteridine Reductase 1 (PTR1) of *L. donovani* (*Ld*) and *L. major* (*Lm*) for *In Silico* Analysis

The X-ray crystallographic structures of Pteridine reductase 1 (PTR1) of *L. major* (*Lm*) and *L. donovani* (*Ld*) were obtained from protein databank (PDB) (Berman et al., 2000; Rose et al., 2017) with IDs 2XOX and 2BFO at resolutions of 2.5 Å and 2.6 Å, respectively. Water molecules, ligands and other hetero atoms, and all chains except chain A were removed using PyMOL (DeLano, 2002).

### Homology Modeling of the Structures of *Ld*GCL, *Lm*GCL, *Ld*TR, and *Lm*TR

Due to the absence of experimentally solved structures of Trypanothione reductase (TR) and Glutamate cysteine ligase (GCL) of *L. donovani* as well as *L. major* in the RCSB PDB database, the primary sequences of Gamma-Glutamylcysteine synthetase (previous name for GCL) of *L. donovani* and Trypanothione reductase (TR) of *L. donovani* as well as *L. major* were obtained from UniProtKB (Magrane and Consortium, 2011) with IDs Q67BG3, C6GKV5, and Q4QJG7, respectively in fasta formats. The sequence of the putative gamma-glutamyl cysteine synthetase of *L. major* (strain Friedlin) with corresponding ID Q4QDM2 was also retrieved from UniProtKB. SWISS-MODEL (accessible *via* https://swissmodel.expasy.org) was used to search for templates of the *Ld*GCL, *Lm*GCL, *Ld*TR, and *Lm*TR (Kiefer et al., 2009).

Modeller 9.2 was then employed to build homology models of *Ld*GCL, *Lm*GCL, *Ld*TR, and *Lm*TR using the most reasonably identical templates with high sequence identity and maximum coverage to the queried sequences (Fiser and Šali, 2003). Homology models were built using pairwise alignment of target and template sequences utilizing spatial restraint techniques with default parameters. The generated models were ranked based on the DOPE scores.

The quality of the modelled structures was assessed using SAVES v5.0 (accessible *via* http://servicesn.mbi.ucla.edu/SAVES/). ERRAT, PROVE, VERIFY, and PROCHECK. ProSA-web were utilised in SAVES v5.0 for quality assessment. Also, Ramachandra plots were generated using Rampage (Sippl, 1993; Wiederstein and Sippl, 2007).

### Binding Site Characterization

The binding pockets were predicted using CASTp (Binkowski et al., 2003; Dundas et al., 2006) and the results were visualized with Chimera 1.12 (Pettersen et al., 2004). The dimensions of the areas and volumes were taken into consideration when selecting the most plausible binding cavities. The active site of the PTR1 proteins was obtained from previous studies (Kaur et al., 2010). Prediction of residues involved in intermolecular bonding was done using LigPLOT+ v1.4.5 (Laskowski and Swindells, 2011).

### Preparation of Ligands and Molecular Docking

The 3D structure of HA was retrieved from PubChem (Wang et al., 2009) with ID 15559629. Docking experiments were performed using AutoDock Vina embedded in PyRx 0.8 (Trott and Olson, 2010; Dallakyan and Olson, 2015). The “make macromolecule” option embedded within PyRx was used to convert the structure into a pdbqt format. The MMFF94 force field and Conjugate Gradients algorithms were used for energy minimization of ligands in 200 steps *via* OpenBabel embedded in PyRx 0.8. Blind docking was carried out on all structures used in this study. The docked structures were visualized and saved as pdb complexes using PyMOL. The resulting docked complexes were imported into LigPLOT+ v1.4.5 (Laskowski and Swindells, 2011) and ligand interaction 2D diagrams were generated for each complex.

### Molecular Dynamics Simulations

The protein-hardwickiic acid complexes were subjected to molecular dynamics simulations (MDs). PRODRG (Schüttelkopf and Van Aalten, 2004) (http://davapc1.bioch.dundee.ac.uk/prodrg/) was used to prepare HA topology prior to the molecular dynamics simulation. Chirality, charges and energy minimization parameters were set to “yes”, “full,” and “no” respectively. MD simulation was conducted with GROMACS 2018 (Van Der Spoel et al., 2005; Abraham et al., 2015) under the GROMOS96 43A1 force field. The box boundary for solvation of the protein-ligand complexes was 1.0 nm. Ions of sodium and chloride were used to neutralize the charges on the complexes. The Steepest Descent was used to minimize the complexes over 1,000 steps.

Thereafter, the complexes were restrained as well as relaxed *via* equilibration. Also, the PME adopted for the simulation was over 100 ns. Finally, graphs were generated using the Xmgrace (Turner, 2005).

### *In Silico* Pharmacokinetic Properties Prediction

HA, miltefosine, amphotericin B and curcumin were subjected to pharmacokinetics profiling using SwissADME. The canonical SMILES of HA (ID: 15559629), miltefosine (ID: 3599), amphotericin B (ID: 5280965), and curcumin (ID: 969516) were retrieved from PubChem and used as inputs for predicting the pharmacokinetic properties *via* SwissADME (Daina et al., 2017).

### Prediction of Leishmanicidal Potential of Hardwickiic Acid

The SMILES file of HA was used to predict the biological activity of the compound (Lagunin et al., 2000; Parasuraman, 2011).

## Results and Discussion

### Structural Elucidation of Hardwickiic Acid (HA)

**Figure 1:**
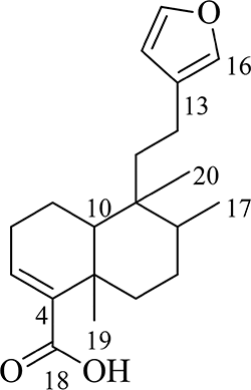
The structure of hardwickiic acid (HA).

Hardwickiic acid (HA) was obtained as a white powder with specific rotation, ${\left[\alpha \right]}_{D}^{20}$ -0.773 (*c* 6.49 x 10^-3^, CHCl_3_). The structure of HA (**Figure 1**) was elucidated from its ^1^H and ^13^C-NMR spectral and MS data as follows: ^1^H NMR (500 MHz, CDCl_3_): 0.77 (3H, Me-20), 0.84 (3H, d, *J* = 6.6 Hz, Me-17), 1.18 (1H, td, *J* = 12.9, 4.0 Hz, H-6a), 1.27 (3H, s, Me-19), 1.39 (1H, d, *J* = 12.1 Hz, 10-H), 1.44 (1H, dd, *J* = 10.1, 4.2 Hz, 7-Ha), 1.47 (1H, m, H-1a), 1.57 (1H, m, 11-Ha), 1.60 (1H, dd, *J* = 7.5, 3.4 Hz, 8-H), 1.66 (1H, dd, *J* = 13.9, 6.3 Hz, 11-Hb), 2.17 (2H, dd, *J* = 13.8, 5.3 Hz, 2-H), 2.23 (1H, m, 12-Ha), 2.31 (1H, t, *J* = 4.9 Hz, 12-Hb), 2.35 (1H, dd, *J* = 10.1, 4.2 Hz, 7-Hb), 2.45 (1H, dt, *J* = 12.9, 3.0 Hz, 6-Hb), 6.26 (1H, s, 14-H), 6.87 (1H, m, 3-H), 7.21 (1H, s, 16-H), 7.35 (1H, s, 15-H); ^13^C NMR (126 MHz, CDCl_3_): 16.1 (C-17, CH_3_), 17.6 (C-1, CH_3_), 18.2 (C-20, CH3), 18.3 (C-12, CH_2_), 18.4 (C-20, CH_3_), 20.7 (C-19, CH_3_), 27.4 (C-7, CH_2_), 27.6 (C-2, CH_2_), 36.0 (C-6, CH_2_), 37.7 (C-5, C), 38.8 (C-9, C), 39.0 (C-11, CH_2_), 46.8 (C-10, CH), 111.1 (C-14, CH), 125.7 (C-13, C), 138.5 (C-16, CH), 140.5 (C-3, C), 141.7 (C-4, C), 142.9 (C-15, CH),173.2 (C-18, C); ESI-MS [M+H]^+^ m/z: 317.1. The MS and ^13^C-NMR spectra of HA are shown in **Supplementary Figures S1** and **S2**, respectively.

### Antipromastigote and Cytotoxicity Activities of HA

**Figure 2:**
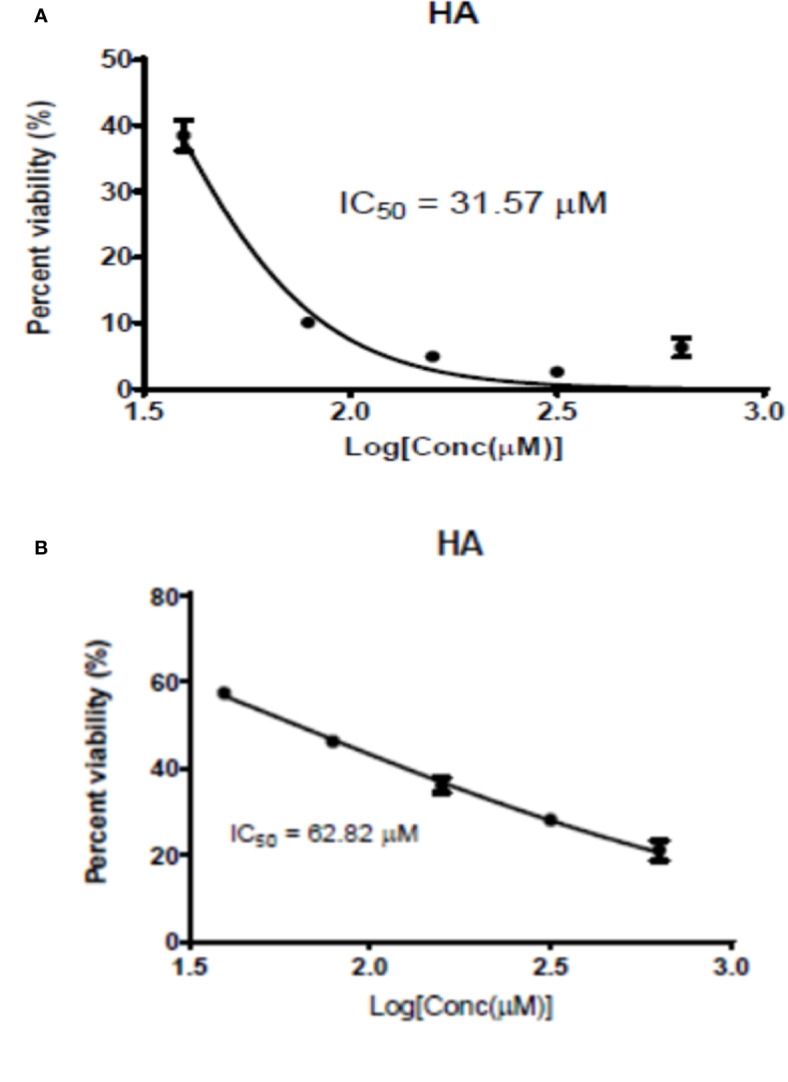
Graphic 1: Dose-response activity of HA on *L. major* **(A)** and on *L. donovani* **(B)**.

The antileishmanial assay of **HA** against promastigotes of *L. major* and *L. donovani* gave IC_50_ values of 62.82 and 31.57 μM, respectively. Moreover, its cytotoxicity on RAW cells was low with a CC_50_ value of 247.83 µM (SI values of 3.94 and 7.85, respectively for *L. major* and *L. donovani*), indicating hardwickiic acid to be a potential antileishmanial compound (**Table 1**, **Figure 2**). There are no previous reports about antileishmanial potential of HA. However, natural products from *Croton* species have been reported to have activity against *Leishmania* parasites. A MeOH : DCM (1:1 v/v) leaf extract from *C. alienus* showed activity on *L. donovani* parasite with an IC_50_ value of 80 μg/ml (Ndunda, 2014). Lima et al. (2015) reported the antileishmanial activity of clerodane diterpenes from *C. cajucara* on *L. amazonensis* parasites (Lima et al., 2015). Trans-dehydrocrotonin was the most potent having IC_50_ of 6.30, 19.18 and 0.47 μg/ml, respectively against promastigotes, axenic and intracellular amastigote forms without toxic effect (CC_50_ >100 μg/ml).

**Table 1:** Antileishmanial and cytotoxicity activities.

	Cytotoxicity on RAW cells	*L. major promastigotes*		*L. donovani promastigotes*	
	CC_50_ (μM)	IC_50_ (μM)	SI	IC_50_ (μM)	SI
Hardwickiic acid	247.83 ± 6.32	62.82 ± 2.53	3.94	31.57 ± 0.14	7.85
Curcumin	29.99 ± 2.82	ND	ND	ND	ND
Amphotericin B	ND	3.67 ± 0.14	ND	3.35 ± 0.14	ND

Concentration of compounds which inhibited parasite proliferation by 50% (IC_50_); concentration of compounds which inhibited normal mammalian cells proliferation by 50% (CC_50_); and not determined (ND).

### Externalization of Phosphatidyl Serine by HA

**Figure 3:**
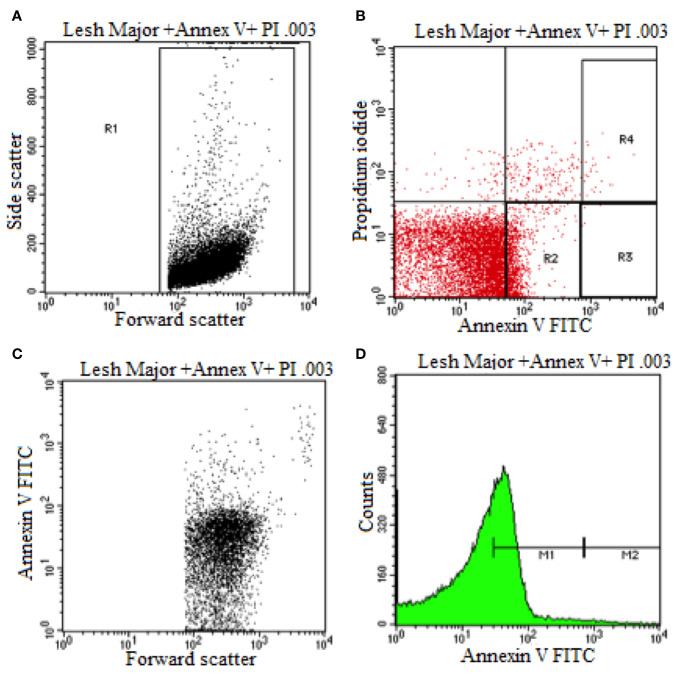
Flow cytometry analysis of promastigotes following treatment with HA and after labeling with annexin-v and pi (untreated Leishmania major promastigote). **(A)** Dot Plot analysis of gated L. Major (R1) showing its size (Forward Scatter) and internal granularity (Side Scatter). **(B)** Dot Plot analysis of cellular apoptotic (Annexin V) and necrotic cells (Propidium Iodide) where L. Major expresses early cellular apoptosis (R2) and necrotic and apoptotic cells (R4). **(C)** Dot Plot analysis of L. Major being expressed in size (Forward Scatter) and cellular apoptosis (Annexin V). **(D)** Histogram plot analysis of L. Major showing low apoptotic cell expression (M1) and high apoptotic cell expression.

Flow cytometry was used to elucidate the *Leishmania* promastigotes parasite death mechanism triggered by HA at its IC_50_ values (**Figure 3**). Untreated control parasites died due to apoptosis or necrosis. Co-staining with annexin V and PI distinguishes between live (PI^−^/annexin V^−^), necrotic or late apoptotic (PI^+^/annexin V^−^) and early apoptotic (annexin V^+^/PI^−^) cells.

During early apoptosis, phosphatidylserine is translocated from the cytosolic face of the plasma membrane to the external face, which can be detected using Annexin V Fluos (Sousa et al., 2014). As shown in **Figure 3** and **Supplementary Figures S3B**, **S4**, and **S5B**, 67.19% and 52.58% of hardwickiic acid-treated *L. major* and *L. donovani* promastigotes, respectively, became annexin positive, indicating early apoptotic phase. In early apoptotic phase, numerous changes occur in the parasite's plasma membrane. Translocation of phophatidylserine from the inside of the parasite membrane to the outside causes changes (Khademvatan et al., 2011). Consequently, 18.84% and 32.52% of a double positive were observed in hardwickiic acid treated-*L. major* and *L. donovani* promastigotes, respectively (**Supplementary Figures S3B** and **S5B**). In contrast, only 1.82% and 16.80% double positive were observed in untreated controls, respectively for *L. major* and *L. donovani* (**Figure 3B** and **Supplementary Figure S3B**). Therefore, HA triggered cell death *via* late apoptotic phase due to membrane integrity loss resulting from the binding of annexin-V and increment in PI incorporation.

### Homology Modeling

The X-ray crystallographic structures of *L. donovani* (*Ld*) and *L. major* (*Lm*) Pteridine reductase 1 (PTR1) with IDs 2XOX and 2BFO, were retrieved from RCSB PDB with resolutions of 2.5 Å and 2.6 Å, respectively (Schüttelkopf et al., 2005; Barrack et al., 2011).

Since there are no solved structures, the sequences of *Ld*TR, *Lm*TR, *Ld*GCL, and *Lm*GCL with corresponding UNIPROT IDs C6GKV5, Q4QJG7, Q67BG3, and Q4QDM2 were retrieved in fasta format, respectively.

PTR1 receptors of *L. major* and *L. donovani* share high sequence conservation with sequence identity of 91% with structurally overlapping active site cavities (Kaur et al., 2010). Using LALIGN (Huang and Miller, 1991) (https://www.ebi.ac.uk/Tools/psa/lalign/), it was shown that *Ld*TR and *Lm*TR shared 95.5% sequence identity and 99.2% similarity, while *Ld*GCL and *Lm*GCL shared 94.8% identity and 98.3% similarity.

A BLAST search for homologues of *Ld*TR, *Lm*TR, and *Ld*GCL was carried out *via* SWISS-MODEL (Schwede et al., 2003; Bienert et al., 2017). The search revealed a total of 5,445 and 5,452 templates that matched the *Ld*TR and *Lm*TR sequences, respectively. The structure with PDB ID: 1TYT was chosen as the reasonable template for comparative modelling of both receptors. 1TYT which is the structure of TR from *Crithidia fasciculata*, is a homo-dimer with a resolution of a 2.6 Å and has sequence similarity of 78.44% and 79.09% to *Ld*TR and *Lm*TR, respectively. *C. fasciculata* is a non-human infective trypanosomatid which is related to *Trypanosoma brucei* and *Leishmania* spp. (Rojas et al., 2014). The BLAST search also revealed a total of 16 and 11 templates as homologues to *Ld*GCL and *Lm*GCL, respectively. The X-ray crystallographic structure of Glutamate cysteine ligase (GCL) from *Saccharomyces cerevisiae* (*Sc*) at resolution of 2.1Å (Biterova and Barycki, 2009) with PDB ID 3IG5 was selected as the reasonably best homologue of both *Ld*GCL and *Lm*GCL with similarity of 39.42% and 39.01%, respectively. The protein encoded by the isolated cDNA of *Lm* is a homologue to those of *S. cerevisiae* and silent information regulator 2 (SIR2) (Yahiaoui et al., 1996).

Modeller 9.2 was used to generate five structures each of *Ld*TR, *Lm*TR, *Ld*GCL, and *Lm*GCL using the selected templates. The most suitable models were selected based on the DOPE scores (**Supplementary Table S1** and **Supplementary Figure S6**). Predicted structures are evaluated using the DOPE score, which is a statistical potential. The model with the least DOPE value can be selected as the best model for the same target (Eswar et al., 2006; Shen and Sali, 2006). The least DOPE scores obtained for the best models of *Ld*TR, *Lm*TR, *Ld*GCL, and *Lm*GCL were -53014.125, -53008.44531, -73083.05469, and -73102.125, respectively.

### Model Quality Assessment

The quality of the selected models of *Ld*TR, *Lm*TR, *Ld*GCL, and *Lm*GCL were assessed using SAVESv5.0. The summary of the quality assessment scores are shown in **Table 2**. ERRAT, PROVE, VERIFY, and PROCHECK were used to assess the quality of the structures. From **Table 2**, *Ld*TR, *Lm*TR, and *Lm*GCL passed VERIFY validation since more than 80% of the amino acids had scores >= 0.2 in the 3D/1D profile, while *Ld*GCL had 77.58%. PROVE showed that *Ld*TR had 83 (4.8%), *Lm*TR had 88 (5.0%), *Ld*GCL had 193 (7.4%), and *Lm*GCL had 210 (8%) buried outlier protein atoms. The overall quality factor obtained *via* ERRAT (**Supplementary Figure S6**) showed that *Ld*TR, *Lm*TR, *Ld*GCL, and *Lm*GCL had overall quality factors of 87.7847, 85.7143, 54.2474, and 57.037%, respectively. *Ld*TR had ERRAT error values at residues 34, 162, 218, 222, 99, 170, 172, and 182. *Lm*TR also had error values at residues 149 to 155, 168 to 173, 175, and 182, while *Ld*GCL and *Lm*GCL showed the most error values, and similar error trends among the four predicted models (**Supplementary Figures S6C, D**).

**Table 2:** Model Evaluation of the Selected Models using various tools.

TOOL	MODEL SCORE			
	*Ld*TR	*Lm*TR	*Ld*GCL	*Lm*GCL
**VERIFY**	90.02%	93.08%	77.58%	80.35%
**ERRAT (Quality Factor)**	87.7847	85.7143	54.2474	57.037
**PROVE**	4.8%	5.0%	7.4%	8.0%
**PROCHECK**	3 Errors, 3 Warnings and 3 Passes.	3 Errors, 3 Warnings and 3 Passes.	4 Errors, 2 Warnings and 3 Passes.	4 Errors, 2 Warnings and 3 Passes.

ProSA-web predicted the z-scores of *Ld*TR, *Lm*TR, *Ld*GCL, and *Lm*GCL as -11.31, -11.81, -7.12, and -8.26, respectively. All four modeled structures were predicted to lie within the z-scores of X-ray determined proteins. The overall quality of the model is shown by the z-score falling within the range known for native proteins of comparable size (Sippl, 1993; Wiederstein and Sippl, 2007).

**Figure 4:**
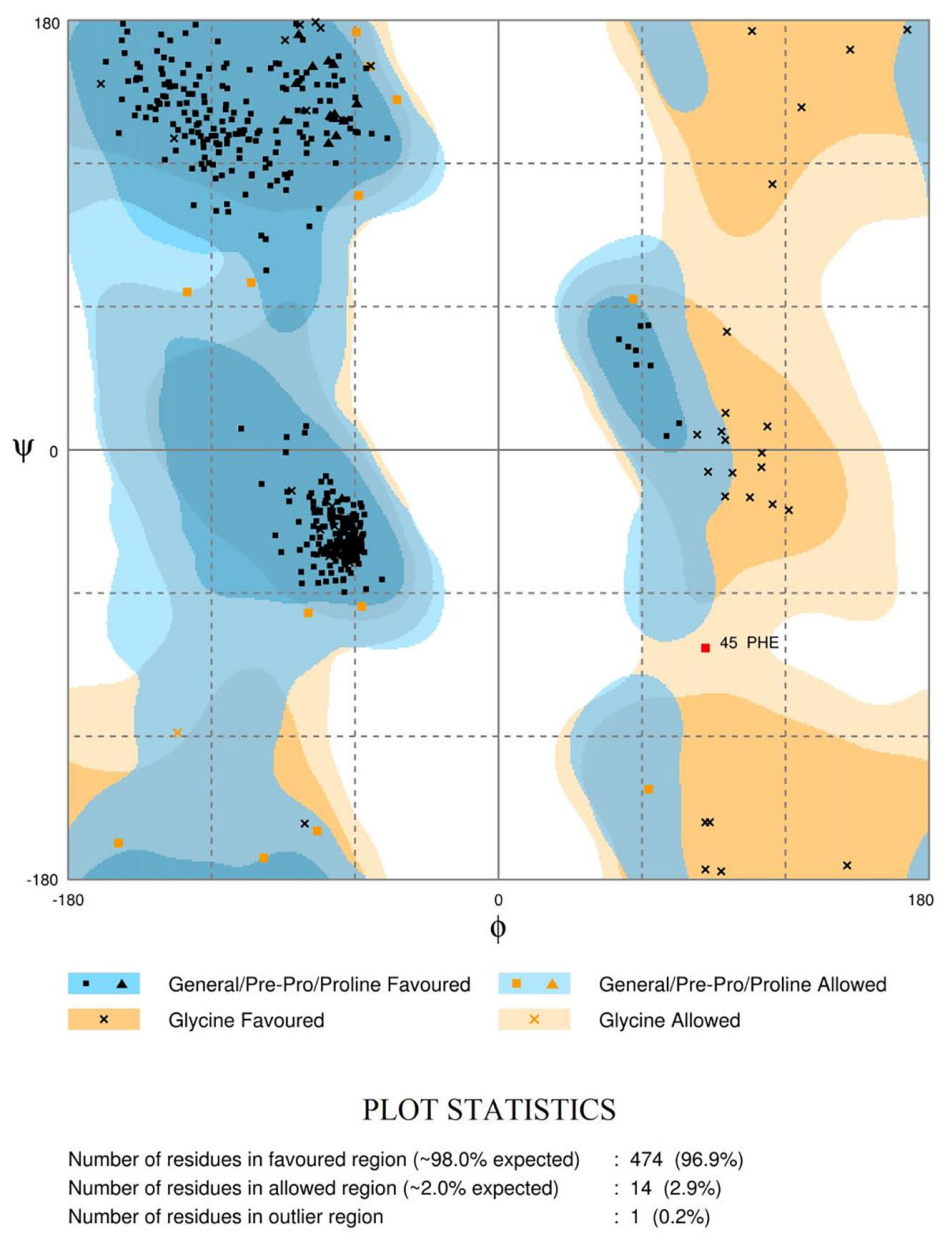
Ramachandran plot of the *Ld*TR modeled receptor obtained *via* RAMPAGE. The plots evaluate the residues in the outlier, allowed and favoured regions.

The Ramachandran plots (**Figure 4** and **Supplementary Figure S7**) evaluate the structural quality of the models using the Rampage (Sippl, 1993; Wiederstein and Sippl, 2007). *Ld*TR had 474 residues (96.9%) in the favoured region, 14 (2.9%) in the allowed region, and only 1 residue (0.2%) in the outlier region. *Lm*TR had 476 residues (97.3%) in the favoured region, 11 (2.2%) in the allowed region and 2 residues (0.4%) in the outlier region. *Ld*GCL had 635 residues (92.7%) in the favoured region, 34 (5.0%) in the allowed region and 16 (2.3%) in the outlier region. *Lm*GCL had 630 residues (92.0%) in the favoured region, 35 (5.1%) in the allowed region and 20 residues (2.9%) in the outlier region. The expected threshold for Rampage is approximately 98% in the favoured region and 2% in the allowed region. All four models had almost all their residues in the favoured regions and were quite close to the expected values. The modeled *Ld*TR had only 1 residue in the outlier region while *Lm*TR had 2 residues in the outlier region. The results from the study showed that the models were of very high quality.

### Binding Site Predictions and Molecular Docking

The active sites of *Ld*PTR1 and *Lm*PTR1 have been determined in previous studies. The active site cavity of *Ld*PTR1 comprise key amino acid residues Arg17, Asn109, Ser111, Asp181, Tyr191, Tyr194, Lys198, Leu226, and Ala230 (Kaur et al., 2010). The receptors of *Lm* and *Ld* PTR1 are highly conserved and have structurally similar active site regions (Cavazzuti et al., 2008; Kaur et al., 2010; Ferrari et al., 2011). The active sites of *Ld*TR, *Lm*TR, *Ld*GCL, and *Lm*GCL were determined using the CASTp. The results were visually analyzed, and the areas and volumes were considered when selecting the most plausible binding sites (**Supplementary Table S2** and **Figure S8**).

HA was docked against six *Leishmania* receptors comprising *Ld*PTR1, *Lm*PTR1, *Ld*TR, *Lm*TR, *Ld*GCL, and *Lm*GCL. From the docking results (**Table 3**), the binding energies of all the six models docked with HA exceeded the threshold of −7.0 kcal/mol (Chang et al., 2007).

**Table 3:** Binding affinities and protein-ligand interactions of HA docked with the six receptors, namely *Ld*PTR1, *Lm*PTR1, *Ld*TR, *Lm*TR, *Ld*GCL, and *Lm*GCL.

Receptor	Binding energy (kcal/mol)	Number of hydrogen bonds	Hydrogen Bond Residues	Hydrogen Bond Length (Å)	Hydrophobic Contacts
*Ld*PTR1	-7.1	4	Lys16, Arg17, Arg17, Ser111.	2.8, 3.05, 3.14, 3.14.	Gly13, Ala15, Lys16, His36, His38, Leu66, Ala110, Ser146, Ser111.
*Lm*PTR1	-7.8	3	Asn109, Gly225, Ser227.	2.9, 2.78, 2.98.	Arg17, Leu18, Gly19, Asn109, Ser111, Phe113, Asp181, Tyr194, Lys198, Pro224, Leu226.
*Ld*TR	-7.6	0	–	–	Gly66, Tyr69, Met70, Leu88, Pro90, Asn91, Thr94, Leu95, Tyr210.
*Lm*TR	-7.5	1	Met70	3.09	Gly66, Ala67, Tyr69, Met70, Ile73, Pro90, Asn91, Thr94, Leu95, Gly209, Tyr210.
*Ld*GCL	-7.4	4	Met1, Gly51, Glu52, Thr330.	3.07, 3.24, 3.05, 3.01.	Met1, Gly51, Glu52, Glu53, Thr101, Pro102, Asp103, Pro105, Thr330, Ile491, Arg494.
*Lm*GCL	-8.0	4	Phe151, Val152, Val152, Cys154.	3.0, 2.99, 3.09, 2.98.	Gln148, Gly149, Asn150, Phe151, Val152, Cys154, Ser155, Asp156, Ser159, Ser164, Leu165, Phe166, Val167, Pro168, Val253, Ser256, Ser257, Arg261.

**Figure 5:**
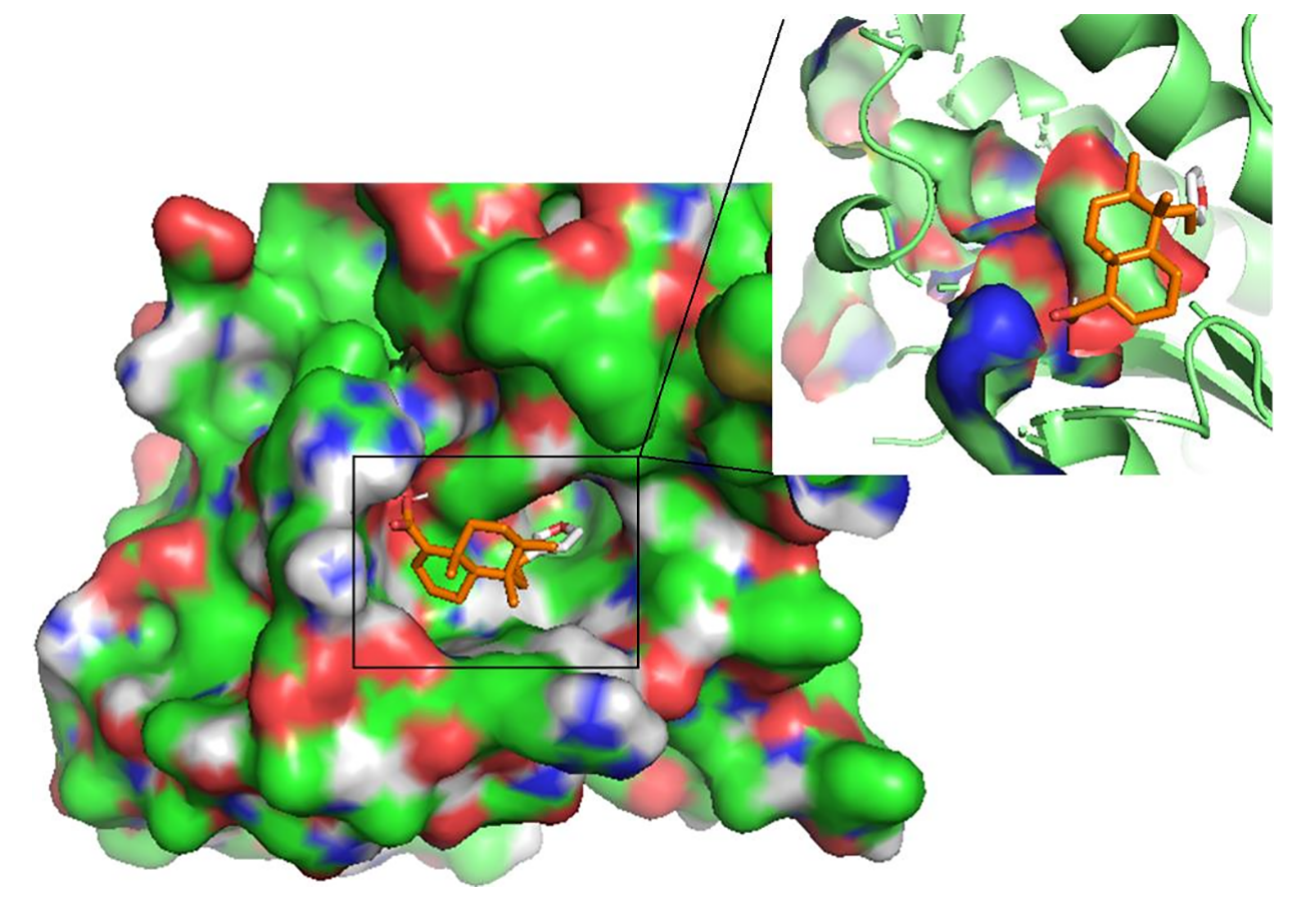
The pose of hardwickiic acid within the binding cavity of *Ld*PTR1. The receptor is represented as a surface while hardwickiic acid as sticks.

HA demonstrated the strongest binding affinity to *Lm*GCL (-8.0kcal/mol), followed by *Lm*PTR1 (-7.8kcal/mol), *Ld*TR (-7.6 kcal/mol), *Lm*TR (-7.5 kcal/mol), *Ld*GCL (-7.4 kcal/mol), and then *Ld*PTR1 (-7.1 kcal/mol). HA is a potential antileishmanial compound based on the docking results which supports the *in vitro* studies conducted herein. The best binding pose of HA in the binding cavities of the 6 proteins are provided (**Figure 5** and **Supplementary Figure S8**). HA docked firmly into the predicted *Lm*TR pocket 4, *Ld*GCL pocket 2, and *Lm*GCL pocket 2.

### Characterization of Protein-Ligand Interactions

**Figure 6:**
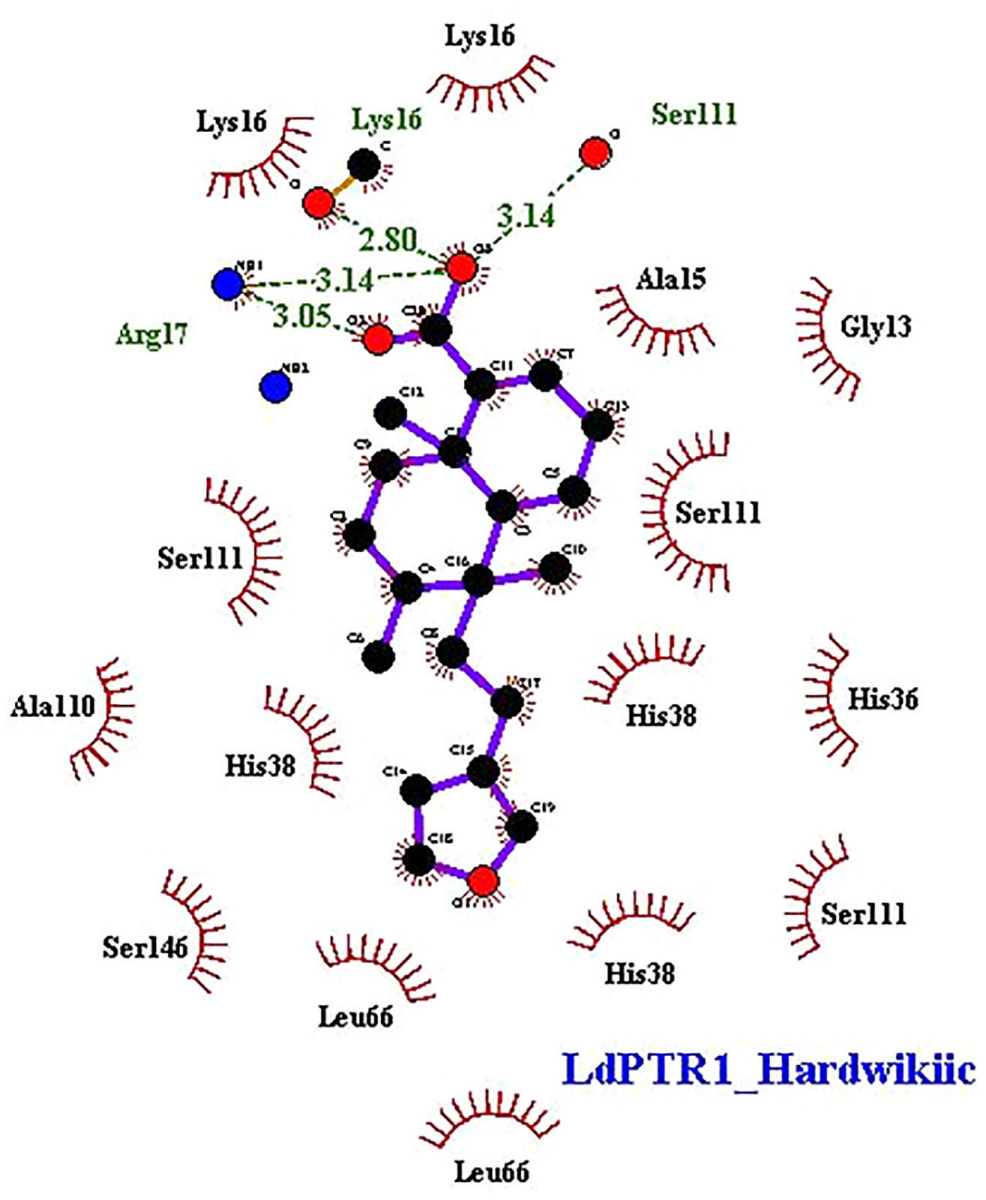
2D representations of protein-ligand interaction between hardwickiic acid and the receptors *Ld*PTR1. Hydrogen bonds are denoted as green dash lines while red spike arcs represent hydrophobic contacts.

The interaction profiles of the receptors and HA complexes have been elucidated (**Table 3** and **Supplementary Table S2**; and **Figure 6**, **Supplementary Figures S8** and **S9**). HA was predicted not to interact *via* H-bonds with *Ld*TR. HA interacted with *Ld*PTR1 *via* hydrogen bonds with Lys16 (2.8 Å bond length), Ser111 (3.14 Å bond length) and Arg17 (bond lengths of 3.05 Å and 3.14 Å). Arg17 and Ser111 were identified as key residues in the active site of PTR1 (Bernal and Coy-Barrera, 2014). HA also interacted with *Lm*PTR1 *via* 3 hydrogen bonds with Asn109, Gly225, and Ser227 with bond lengths of 2.9 Å, 2.78 Å, and 2.98 Å, respectively. Some dimeric xanthanolide compounds were shown to have the best binding affinity when docked against PTR1. Also, the dimeric xanthanolide compounds formed hydrogen bond interactions with Ser111, Ser227, and Arg17, which are critical amino acid residues required for stability (Bernal and Coy-Barrera, 2014). These two compounds were reported to be reasonably active against *L. donovani* amastigotes (IC_50_:22–27 µM) (Schmidt et al., 2009). Herein, hardwickiic acid is a promising lead which warrants further structural optimization studies for PTR1 inhibition.

HA also interacted with *Lm*TR *via* a hydrogen bond with Met70 with bond length of 3.09 Å. Similarly, HA interacted with Met1, Gly51, Glu52, and Thr330 of *Ld*GCL with bond lengths of 3.07 Å, 3.24 Å, 3.05 Å, and 3.01 Å, respectively. It was also observed that HA interacted with *Lm*GCL *via* 4 hydrogen bonds with Phe151, Val152, Val152, and Cys154 of lengths 3.0 Å, 2.99 Å, 3.09 Å, and 2.98 Å, respectively. The high numbers of hydrogen bonds with relatively short bond distances indicate a strong binding which thereby positions HA as a promising leishmanicide. HA shared a single hydrophobic contact with Ser111 in *Ld*PTR1, and two with Arg17 and Ser111 in *Lm*PTR1.

### Molecular Dynamics Simulation of Complexes

**Figure 7:**
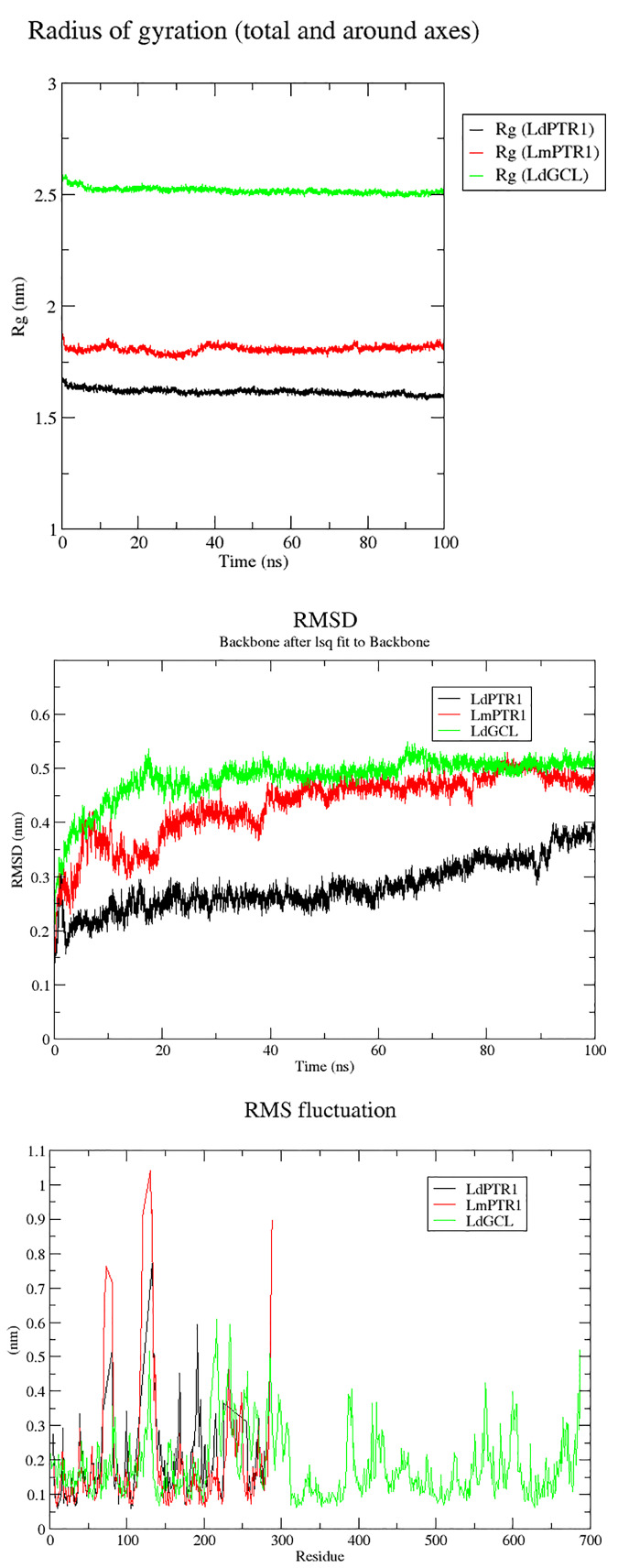
*Rg*, RMSDs and RMSFs plots for the protein–hardwickiic acid complex MDs undertaken in 100 ns: **(A)** *Rg* plots of the three proteins in complex with hardwickiic acid. **(B)** The plot of RMSDs over 100 ns for the three complexes. **(C)** RMS Fluctuations (RMSFs) of amino acid residues pertaining to the three proteins in complex with hardwickiic acid. *Ld*PTR1, *Lm*PTR1, and *Ld*GCL complexes are illustrated as black, red, and green colors, respectively.

Molecular dynamics simulations over the course of 100 ns were carried out on *Ld*PTR1, *Lm*PTR1 and *Ld*GCL complexed with hardwickiic acid. The stability of the protein-ligand complexes was determined over the 100 ns simulations. The radius of gyration (*R_g_*) which determines its compactness was generated for all the three complexes. A protein structure which is stably folded maintains a fairly steady *R_g_*, while the *R_g_* of an unfolded protein changes significantly over a period. From the *R_g_* plots (**Figure 7A**), all the three protein-ligand complexes were very stable during the entire simulation. All systems were compact, with *Ld*PTR1 having the least *R_g_* of 1.65 nm. *Lm*PTR1 had an average *R_g_* of 1.8 nm, and *Ld*GCL had the lowest average *R_g_* of 2.5 nm. *Ld*GCL demonstrated the most stability with little fluctuations observed around 0 to 8 ns. *Lm*PTR1 showed the least stability among the three complexes, fluctuated from 0 to about 40 ns, then stabilized thereafter.

The RMSDs were computed to determine the stability of the complexes which is shown by fluctuations. During the equilibration phase, RMSDs of the complexes increased and then converged 20 ns later. From **Figure 7B**, the three complexes (backbone) showed stability after a gradual rise. The *Ld*PTR1-hardwickiic acid complex increased in RMSD from 0 ns until about 20 ns, after which it appeared fairly stable. After about 40 ns, the RMSD of *Lm*PTR1-HA complex averaged around 0.45 nm. The RMSD of *Ld*GCL-HA complex was the most stable form. It was observed to rise during the equilibration phase from 0.2 nm to about 0.5 nm during the first 20 ns.

To determine protein regions which exhibited higher structural flexibility, the root mean square fluctuations (RMSF) per residue was determined (**Figure 7C**). *Ld*PTR1 and *Lm*PTR1 showed similar trends by exhibiting greater fluctuations with the highest RMSFs. This corroborated the high structural identity of the two protein structures. For both *Ld*PTR1 and *Lm*PTR1, the high fluctuation regions were within residue index 60 to 80 and 100 to 140 with 100 to 140 being the highest. *Ld*PTR1 also exhibited high fluctuation from residues 180 to 200. The active sites of both PTR1 proteins were around residue index 100 to 140 and 180 to 200. It was highly possible that key residues Asn109 and Ser111 caused the fluctuations from 100 to 140, while Asp181, Tyr191, Tyr194, and Lys198 were responsible for the fluctuations from 180 to 200. *Ld*GCL demonstrated the lowest RMSF values as compared to *Ld*PTR1 and *Lm*PTR1. The major fluctuations for *Ld*GCL were observed in the regions with residue index 105 to 140 and 200 to 230. It could be possible that Thr101, Pro102, Asp103, and Pro105 which had contacts with hardwickiic acid were responsible for the high spike exhibited around residues 105 to 140.

### *In Silico* Pharmacokinetic Properties Prediction

All the four compounds were predicted to have moderately soluble with low ESOL logS (**Table 4**). ESOL logS quantifies the aqueous solubility of a molecule (Daina et al., 2017). HA and curcumin were predicted to possess good gastrointestinal (GI) absorption implying a high possibility of enhanced absorption of orally administered drugs into the intestinal tract and the bloodstream (Kwofie et al., 2019). The Abbot Bioavailability scores of the compounds were also determined. HA was predicted to possess the highest value (0.56), whereas amphotericin B was predicted to possess the lowest (0.17). Both miltefosine and curcumin had a bioavailability score of 0.55. The Abbot Bioavailability score denotes oral bioavailability of at least 10% in rat or measurable permeability in Caco-2. It relies on total charge, and topological polar surface area (TPSA) together with violations of Lipinski's rules to classify compounds into four categories with probabilities of 11%, 17%, 56%, or 85% (Martin, 2005).

**Table 4:** Prediction of pharmacokinetic properties for HA, miltefosine, amphotericin B and curcumin using SwissADME.

Compound	Molecular Weight	ESOL LogS	ESOL Class	GI Absorption	BBB Permeant	Pgp substrate	Bioavailability score
HA	316.4	-5.25	Moderately soluble	High	Yes	No	0.56
Miltefosine	407.6	-5.32	Moderately soluble	Low	No	Yes	0.55
Amphotericin B	924.1	-5.37	Moderately soluble	Low	No	Yes	0.17
Curcumin	368.4	-3.94	Moderately soluble	High	No	No	0.55

Properties determined include P-glycoprotein substrate (Pgp), Gastrointestinal absorption (GI), Blood Brain Barrier permeation (BBB), Estimated Solubility (ESOL), and Bioavailability score.

Since HA and curcumin were also predicted as non-substrate of P-gp, they can be distributed effectively within the circulatory system (**Table 4**). The P-glycoprotein (P-gp) acts as physiological filter by eliminating unwanted materials including toxins (Lin and Yamazaki, 2003). The Blood Brain Barrier (BBB) permeation denotes the effectiveness of molecules to permeate the barrier to initiate signaling after binding to key receptors. Also, a drug must permeate this barrier in order to have pharmacological effect on the brain parenchyma (Suenderhauf et al., 2012). Only Hardwickiic acid was predicted to have the ability to permeate the barrier, (**Table 4**).

### Biological Activity Prediction for Hardwickiic Acid

The activity of HA was predicted using features of molecules with known activities by implementing a Bayesian algorithm (Parasuraman, 2011). Biological activity is based on the predicted probable inactivity (Pi) and probable activity (Pa) of a molecule (Jamkhande et al., 2016). PASS predicted HA to be Caspase-3 stimulant with Pa 0.757 and Pi 0.008; and Caspase-8 stimulant with Pa 0.688 and Pi 0.004. HA was also predicted to possess antiprotozoal activity (specifically, antileishmanial) with Pa of 0.434 and Pi 0.037. From recent studies, *Leishmania* infections have been reported to delay host cell apoptosis by inhibiting caspase-3 activity (Ruhland et al., 2007; Abhishek et al., 2018). Caspase-8 inhibition following *L. major* infection has also been shown to decrease the expression of interferon gamma by CD4 and CD8 T cells (Pereira et al., 2008). Therefore, HA maybe implicated in the apoptotic mechanisms. For a given compound activity, whenever the Pa > Pi, the predicted biological activity can be pursued experimentally (Jamkhande and Barde, 2014; Kwofie et al., 2018). Therefore, HA is an attractive antileishmanial candidate, which reinforces the results of the *in vitro* studies.

## Conclusion

The study evaluated the leishmanicidal potential of HA characterised from the stembark of *Croton sylvaticus* against *Leishmania donovani* and *L. major* promastigotes. HA exhibited strong antileishmanial activity on *L. donovani* promastigotes with an IC_50_ of 31.57 ± 0.06 µM when compared to amphotericin B with an IC_50_ of 3.35 ± 0.14 µM. Also, the cytotoxic activity of CC_50_ = 247.83 ± 6.32 µM was obtained against 29.99 ± 2.82 µM for curcumin with selectivity index of SI = 7.85. Molecular modelling and docking studies revealed that HA had binding affinity greater than -7.0 kcal/mol with all 6 *Leishmania* receptors used in this study. HA exhibited the highest binding affinity (-8.0 kcal/mol) with *Lm*GCL. HA was also observed to interact with critical residues Lys16, Ser111 and Arg17 required for *Ld*PTR1 binding. Moreover, HA was predicted as Caspase-3 and Caspase-8 stimulants and possesses antileishmanial activity. Therefore, HA may play a critical role in caspase mediated apoptotic mechanisms. The potential apoptotic role was further corroborated experimentally since HA was shown to induce parasite death by loss of membrane integrity. HA is an attractive antileishmanial candidate worthy of further *in vivo* experimentation.

## Data Availability Statement

All datasets generated for this study are included in the article/**Supplementary Material**.

## Author Contributions

DO-S, LY, and SK conceptualised the project. DO-S, JC, and BA were responsible for isolation and characterisation of HA. LY and JT conducted the biological assays while SK and EB undertook the cheminformatics. All authors co-wrote the first draft of the manuscript and proofread the submitted manuscript.

## Funding

Bill and Melinda Gates Foundation supported the postdoctoral fellowship (OPP52155) of LRTY at NMIMR, Ghana. This work was supported by funds from a World Bank African Centres of Excellence grant (ACE02-WACCBIP: Awandare) and a DELTAS Africa grant (107755/Z/15/Z: Awandare). The DELTAS Africa Initiative is an independent funding scheme of the African Academy of Sciences (AAS)’s Alliance for Accelerating Excellence in Science in Africa (AESA) and supported by the New Partnership for Africa’s Development Planning and Coordinating Agency (NEPAD Agency) with funding from the Wellcome Trust [107755/Z/15/Z: Awandare] and the UK government. The views expressed in this publication are those of the author(s) and not necessarily those of AAS, NEPAD Agency, Wellcome Trust or the UK government.

## Conflict of Interest

The authors declare that the research was conducted in the absence of any commercial or financial relationships that could be construed as a potential conflict of interest.
