# Supplementary material for: Leishmanicidal Potential of Hardwickiic Acid Isolated From Croton sylvaticus
Source: Front Pharmacol. 2020 May 25;11:753. doi: 10.3389/fphar.2020.00753 (PMC7261830; doi:10.3389/fphar.2020.00753)
Supplement: Supplementary file 1 [file DataSheet_1.docx]

**SUPPLEMENTARY MATERIAL**

## **Leishmanicidal potential of hardwickiic acid isolated from *Croton sylvaticus***

Crentsil Justice Afrifa^1^, Lauve Rachel Tchokouaha Yamthe^2,3,4^, Barbara Zenabu Anibea^1^, Emmanuel Broni^5^, Samuel Kojo Kwofie^5,6,7,8*^, John Kweku Amissah Tetteh^9^, Dorcas Osei-Safo^1^

^1^Chemistry Department, School of Physical and Mathematical Sciences, College of Basic and Applied Sciences (CBAS), University of Ghana, P. O. Box LG 56, Legon, Accra, Ghana

^2^Institute for Medical Research and Medicinal Plants Studies, P.O. Box 13033, Yaoundé, Cameroon

^3^Department of Parasitology, Noguchi Memorial Institute for Medical Research, College of Health Sciences, University of Ghana, LG581, Legon, Accra, Ghana

^4^Antimicrobial and Biocontrol Agents Unit, University of Yaoundé 1, P. O. Box 812, Yaoundé, Cameroon

^5^Department of Biomedical Engineering, School of Engineering Sciences, CBAS, University of Ghana, PMB LG77, Legon, Accra, Ghana

^6^West African Center for Cell Biology of Infectious Pathogens, Department of Biochemistry, Cell and Molecular Biology, CBAS, University of Ghana, LR54, Accra, Ghana

^7^Department of Medicine, Loyola University Medical Center, Maywood, IL 60153, USA

^8^ Department of Physics and Engineering Science, Coastal Carolina University, Conway, SC 29528

**^9^Department of Immunology, Noguchi Memorial Institute for Medical Research, College of Health Sciences, University of Ghana, LG581, Legon, Accra, Ghana**

**Table of Contents**

**Figure S1**. MS of **HA**

**Figure S2**. ^13^C-NMR spectrum (126 MHz, CDCl_3_) of **HA**

**Figure S3:** Flow cytometry analysis of promastigotes following treatment with HA and after labelling with annexin-v and pi (compound treated L. major promastigote).

**Figure S4:** Flow cytometry analysis of promastigotes following treatment with HA and after labelling with annexin-v and pi (untreated L. donovani promastigote).

**Figure S5**: Flow cytometry analysis of promastigotes following treatment with HA and after labelling with annexin-v and pi (compound treated L. donovani promastigote).

Figure S6: ERRAT Quality Plots of the selected models: (A) *Ld*TR III, (B) *Lm*TR I, (C) *Ld*GCL V and (D) *Lm*GCL. Green peaks show the passed values, yellow signifies the regions with warning and red peaks represent the regions with error values.

Figure S7: Ramachandran plot of the 4 modelled receptors obtained via RAMPAGE: (A) *Lm*TR I, (B) *Ld*GCL V and (C) *Lm*GCL. The plots evaluate the number of residues in the favoured, allowed and outlier regions.

Figure S8: The binding poses of hardwikiic acid in the binding cavities of *Lm*PTR1 (A), *Ld*TR III (B), *Lm*TR I (C), *Ld*GCL (D) and *Lm*GCL (E). The receptors are represented as a surface whiles hardwickiic acid as sticks.

Figure S9: 2D representations of Protein-ligand interaction between hardwickiic acid and the receptors: (A) *Lm*PTR1, (B) *Ld*GCL V and (C) *Lm*GCL V. Hydrogen bonds are shown with green dash lines and hydrophobic contacts are shown as red spike arcs.

Table S1: Dope Scores of Generated Models of *Ld*TR, *Lm*TR, *Ld*GCL and *Lm*GCL. The models with the least DOPE scores were selected as the plausible structures.

**Table S2**: Predicted binding sites for the 4 modelled receptors using CASTp. The sizes of the volumes, areas and residues lining the pockets are provided

**Figure S1**. MS of **HA**

**
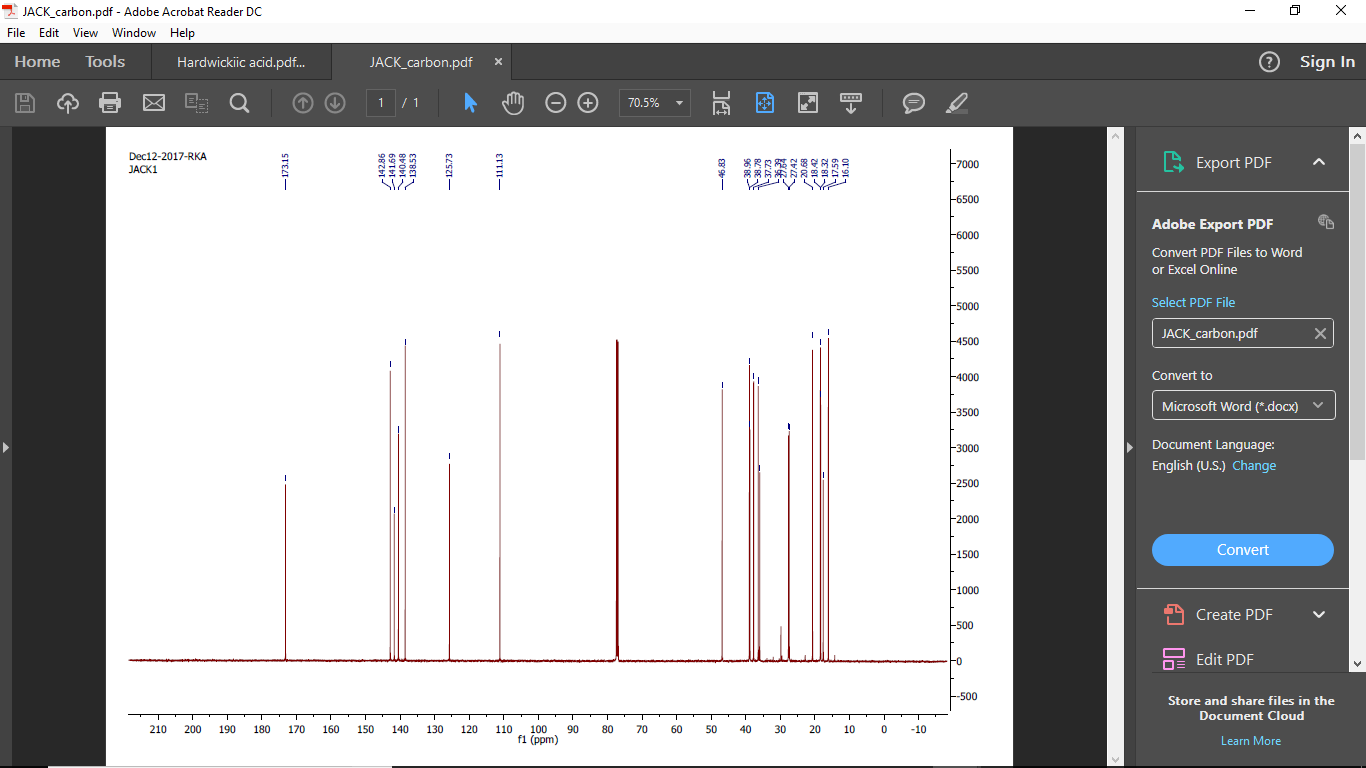
Figure S2**. ^13^C-NMR spectrum (126 MHz, CDCl_3_) of **HA**

**
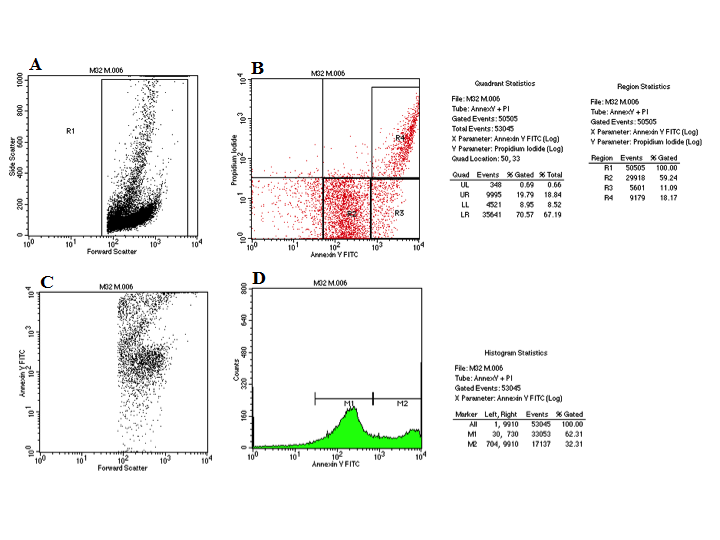
**

Figure S3: Flow cytometry analysis of promastigotes following treatment with HA and after labeling with annexin-v and pi (compound treated L. major promastigote).

**
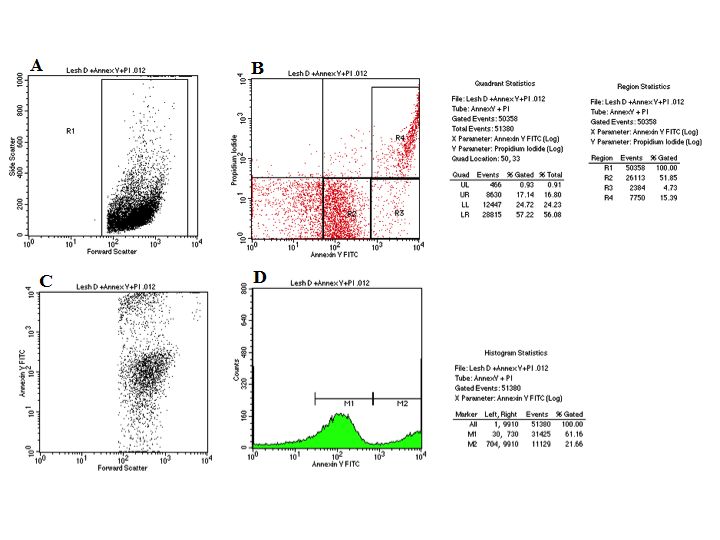
**

Figure S4: Flow cytometry analysis of promastigotes following treatment with HA and after labeling with annexin-v and pi (untreated L. donovani promastigote).

**
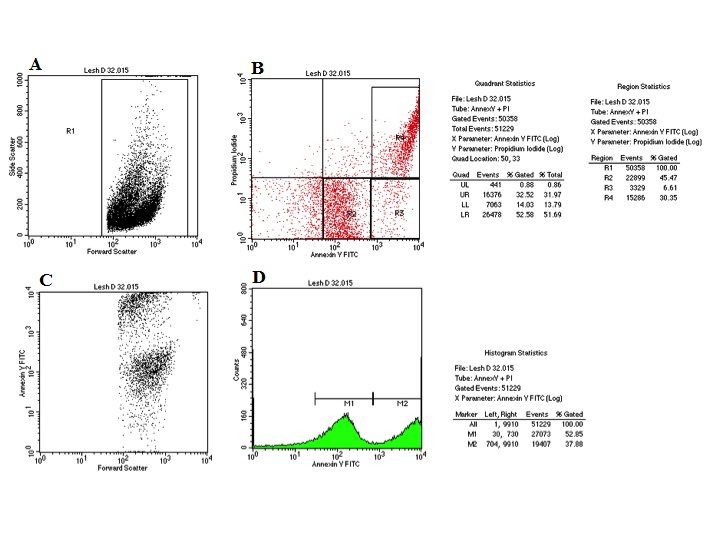
**

Figure S5: Flow cytometry analysis of promastigotes following treatment with HA and after labeling with annexin-v and pi (compound treated L. donovani promastigote).

**A**


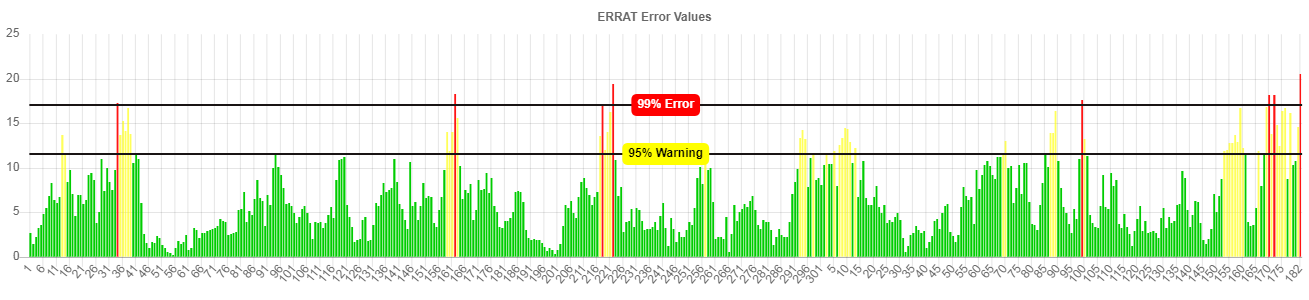


**B**


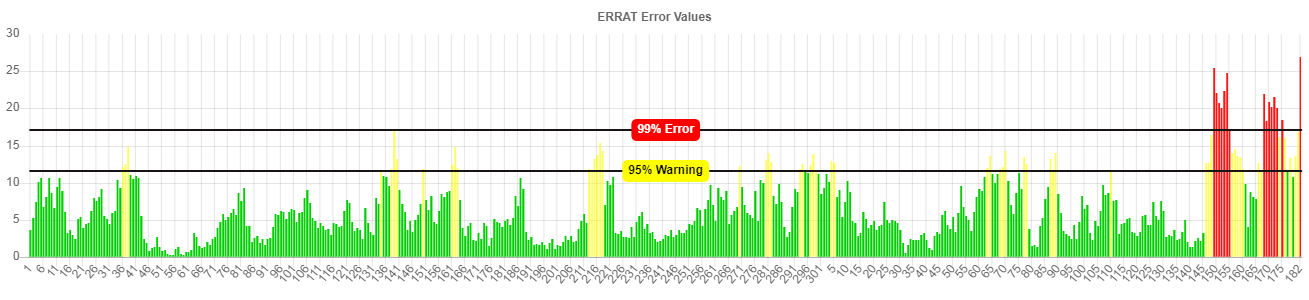


**C**


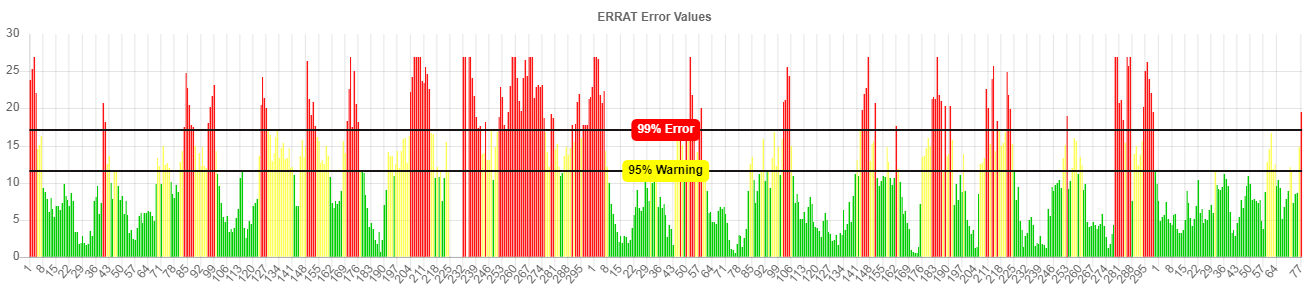


**D**

**
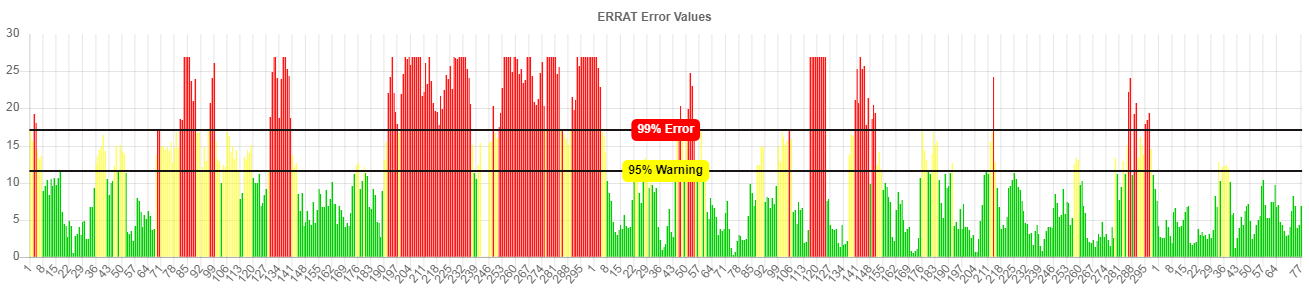
**

Figure S6: ERRAT Quality Plots of the selected models: (A) *Ld*TR III, (B) *Lm*TR I, (C) *Ld*GCL V and (D) *Lm*GCL. Green peaks show the passed values, yellow signifies the regions with warning and red peaks represent the regions with error values.

# A


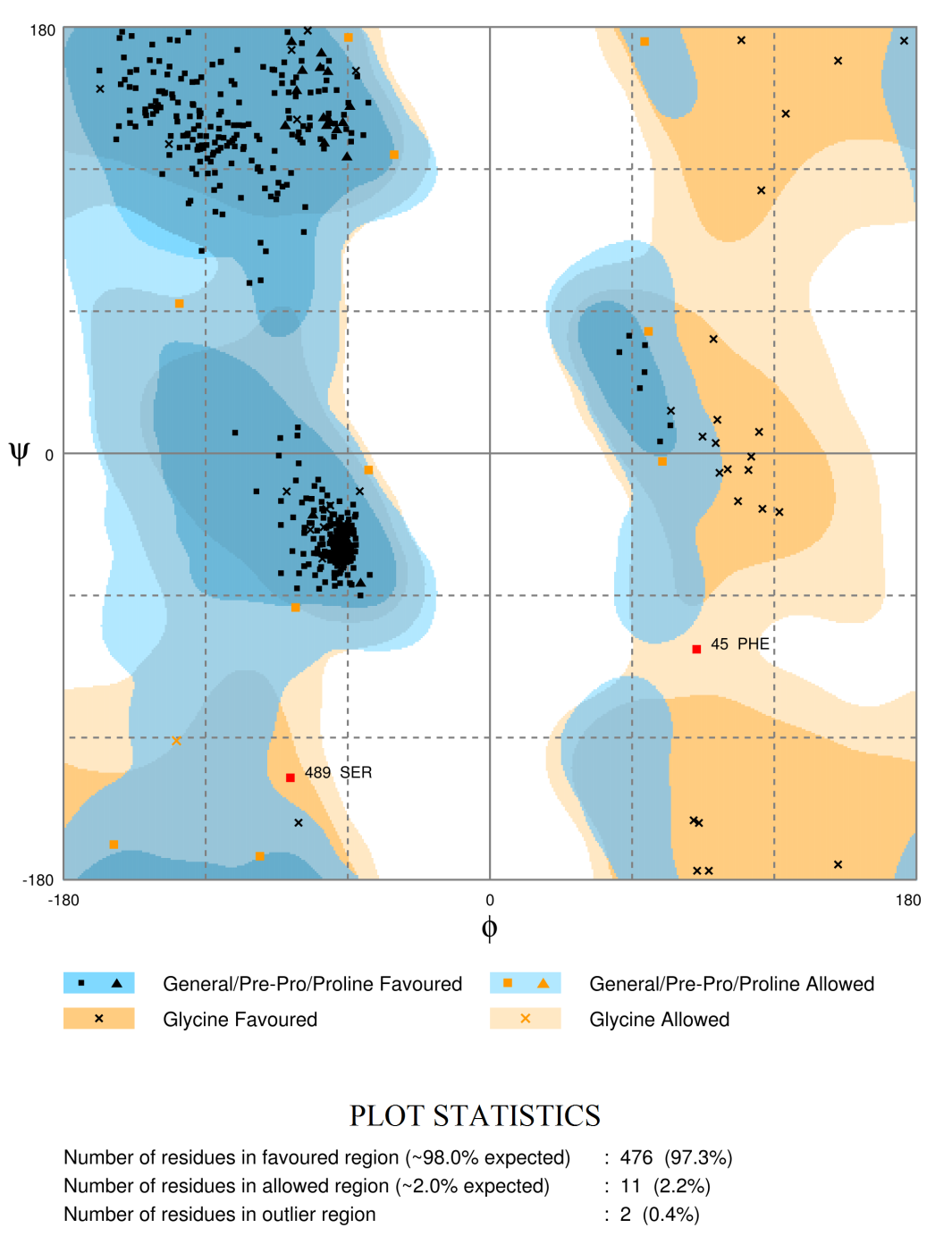


# B

#
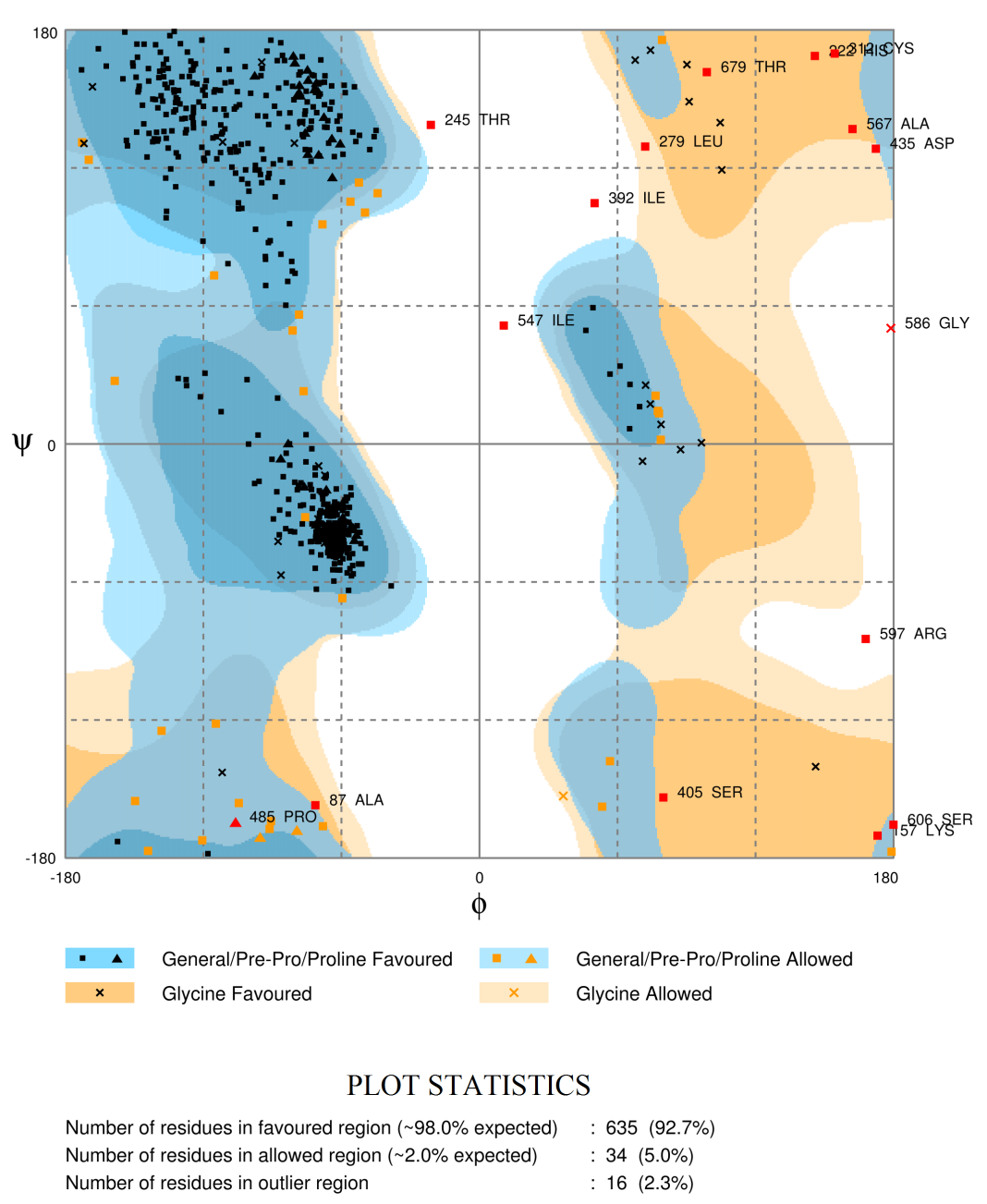


**C**

**
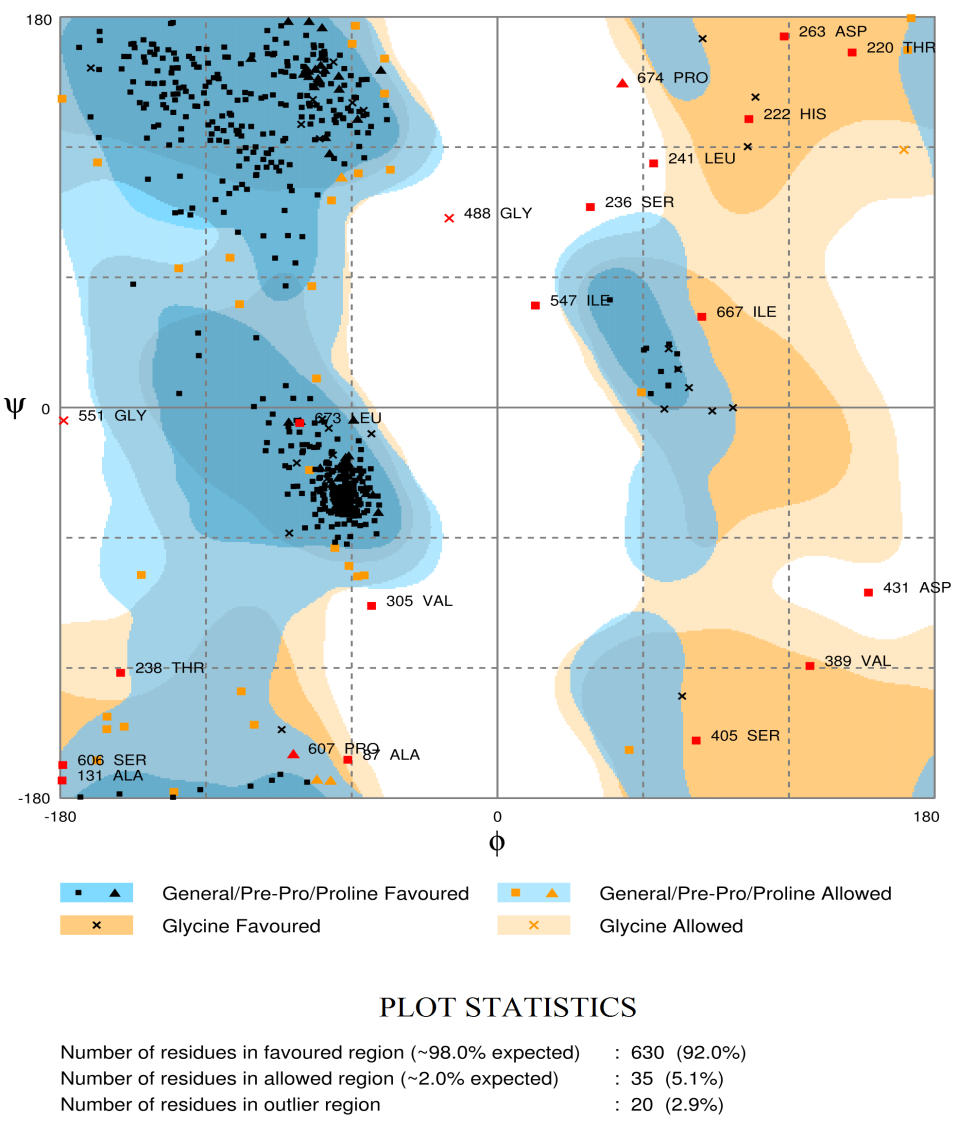
**

Figure S7: Ramachandran plot of the 4 modelled receptors obtained via RAMPAGE: (A) *Lm*TR I, (B) *Ld*GCL V and (C) *Lm*GCL. The plots evaluate the number of residues in the favoured, allowed and outlier regions.

**A LmPTR1**

**
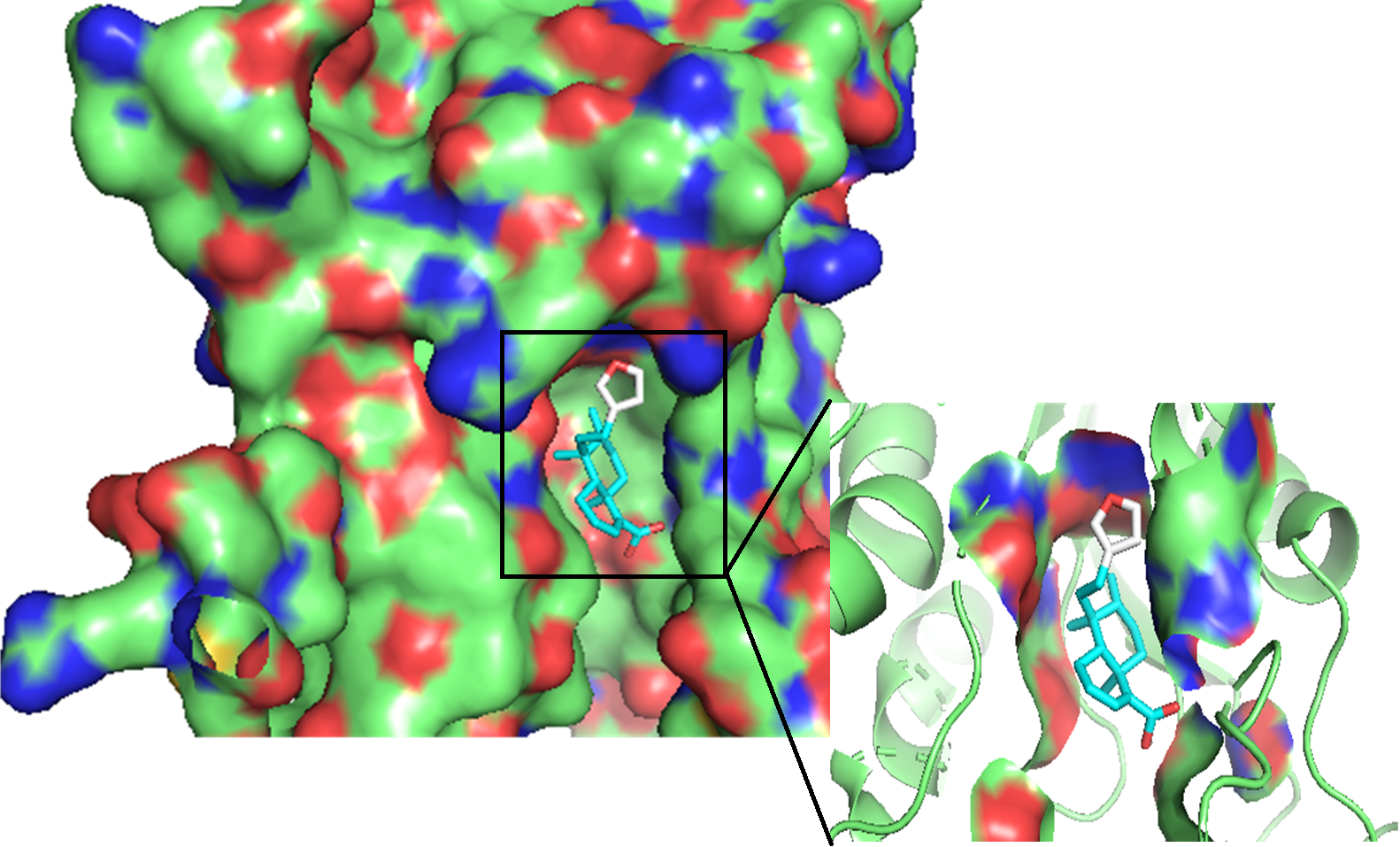
**

**B LdTR III**

**
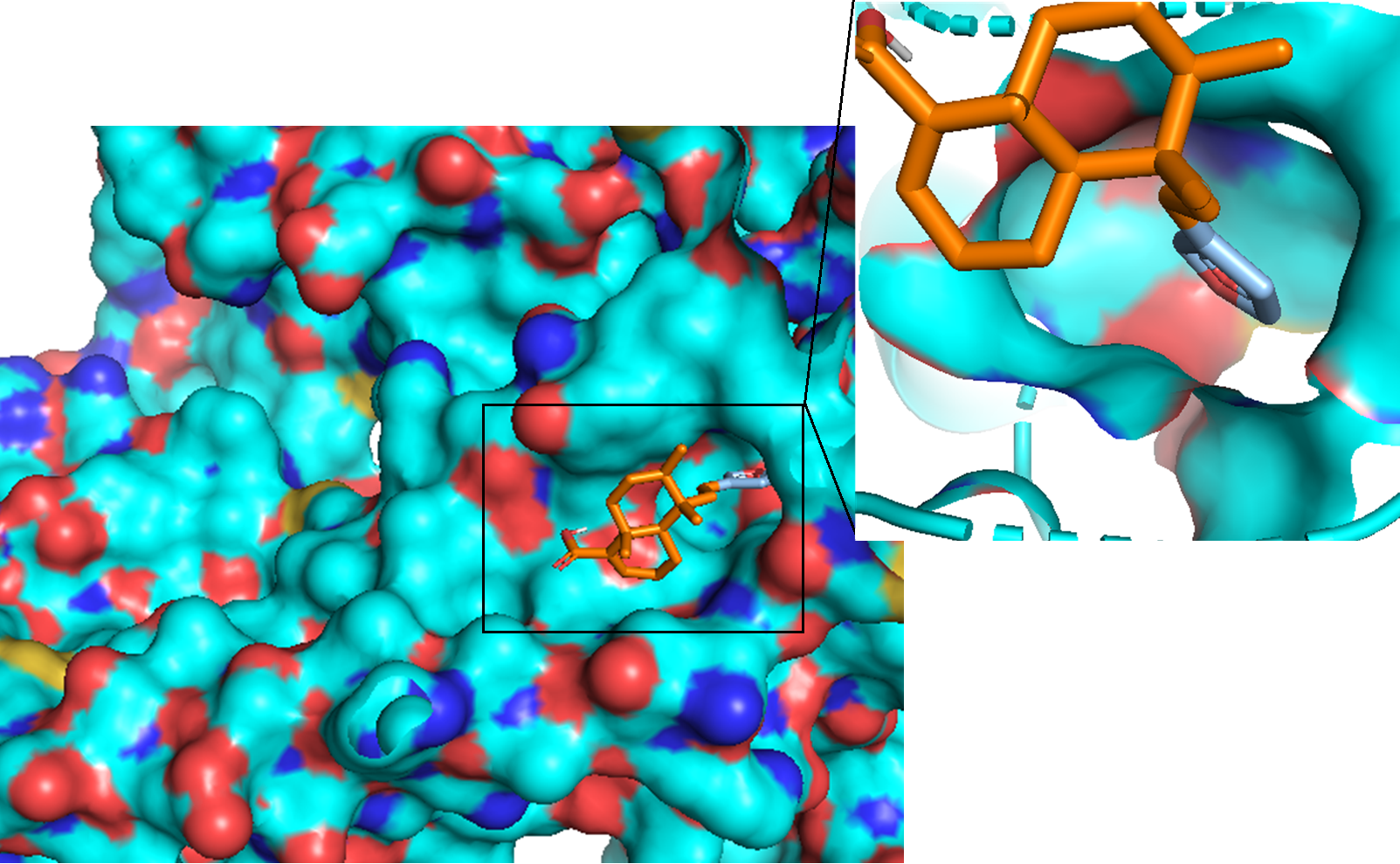
**

**C LmTR I**

**
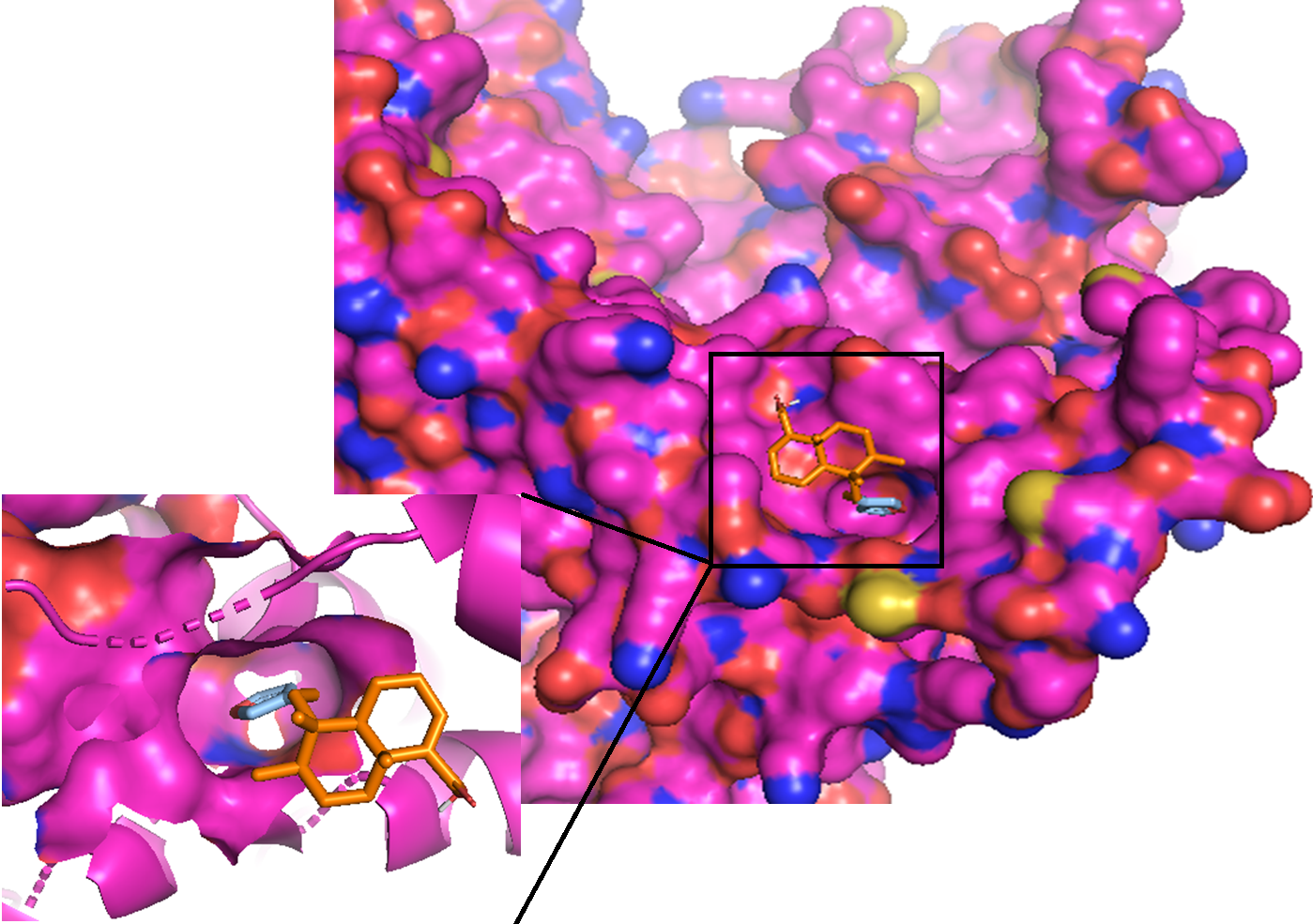
**

**D LdGCL
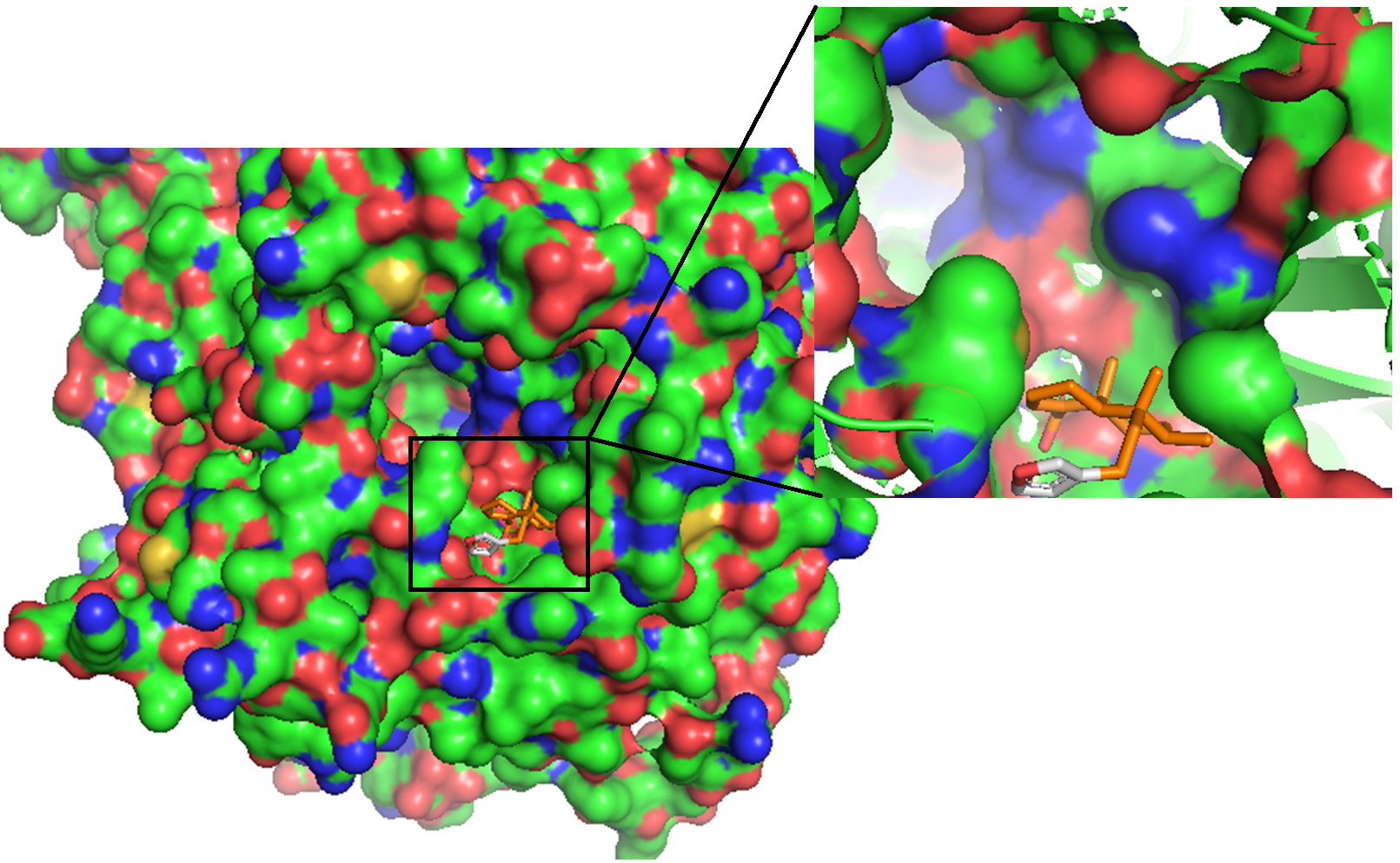
**

**E *Lm*GCL**

**
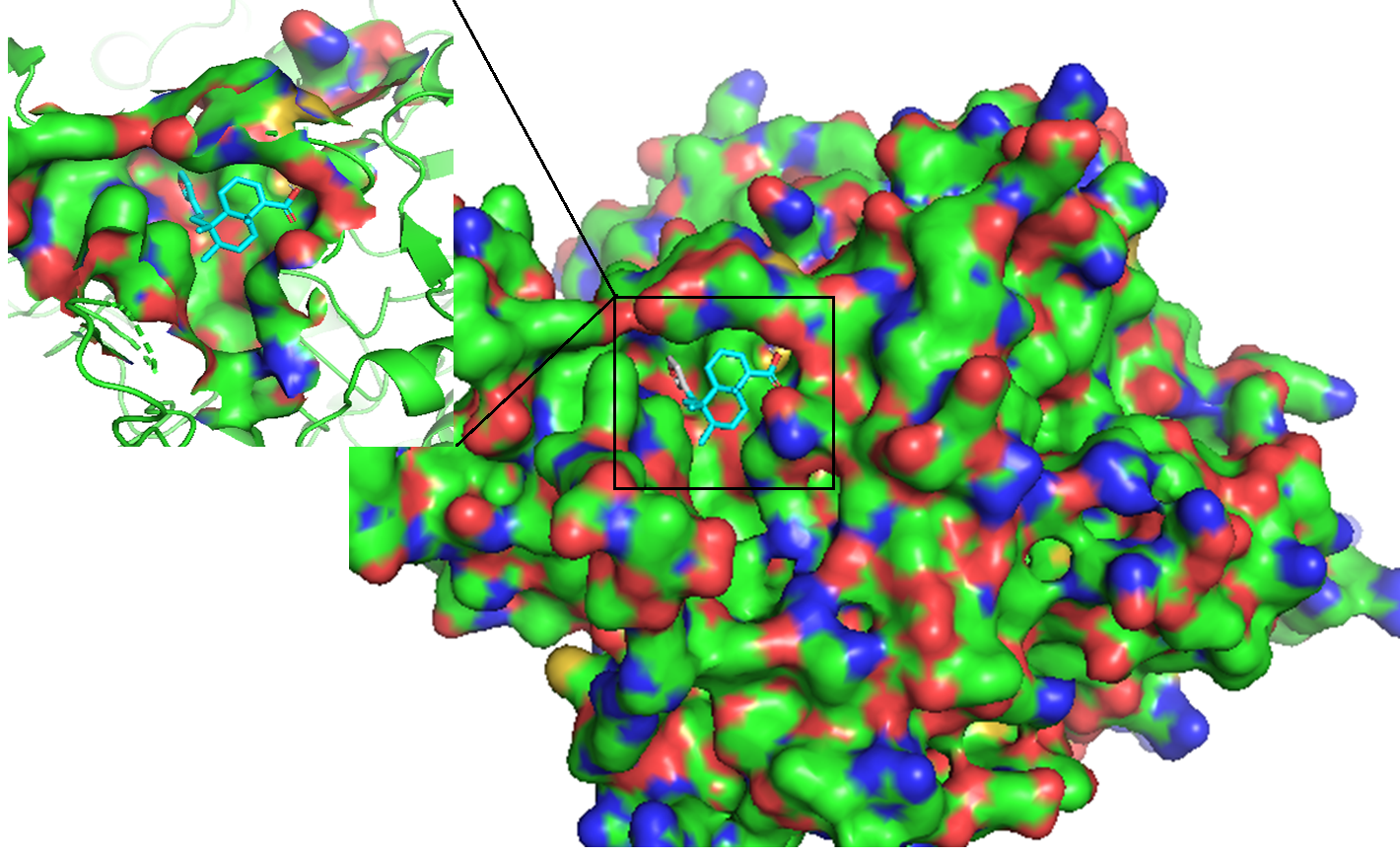
**

Figure S8: The binding pose of hardwikiic acid in the binding cavities of *Lm*PTR1 (A), *Ld*TR III (B), *Lm*TR I (C), *Ld*GCL (D) and *Lm*GCL (E). The receptors are represented as a surface whiles hardwickiic acid as sticks.

**A**


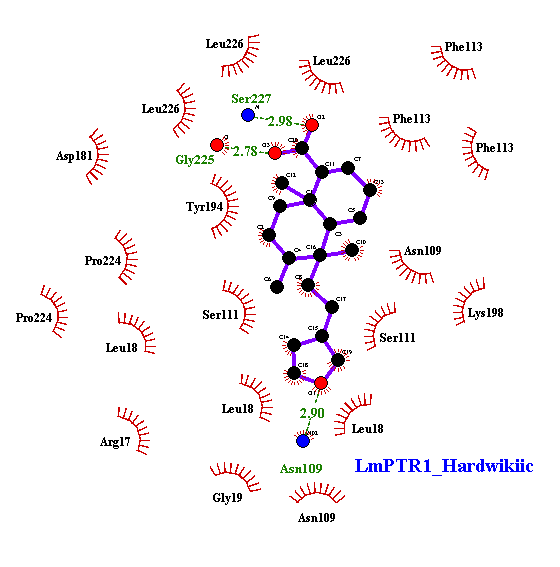


**B**

**
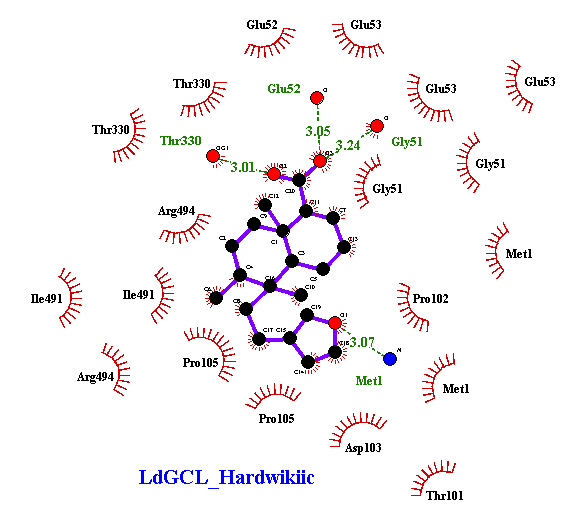
**

**C**

**
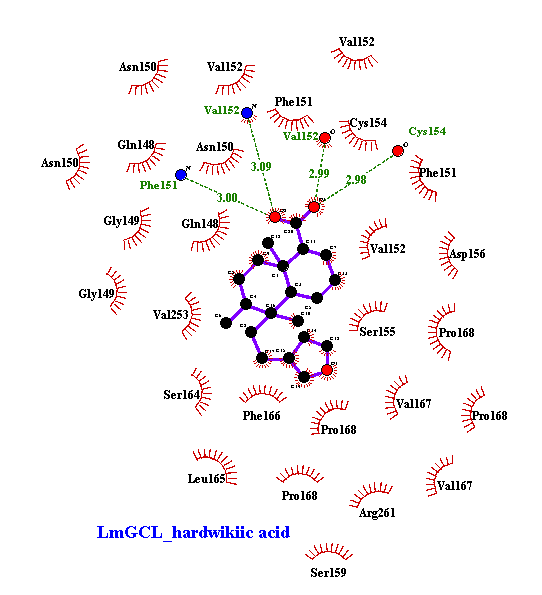
**

Figure S9: 2D representations of Protein-ligand interaction between hardwickiic acid and the receptors: (A) *Lm*PTR1, (B) *Ld*GCL V and (C) *Lm*GCL V. Hydrogen bonds are shown with green dash lines and hydrophobic contacts are shown as red spike arcs.

Table S1: Dope Scores of Generated Models of *Ld*TR, *Lm*TR and *Ld*GCL. The models with the least DOPE scores were selected as the plausible structures.

| **SCORES** | **MODELS** | | | | |
| --- | --- | --- | --- | --- | --- |
| ***L. donovani* Trypanothione reductase (*Ld*TR)** | | | | | |
|  | ***Ld*TR I** | ***Ld*TR II** | ***Ld*TR III** | ***Ld*TR IV** | ***Ld*TR V** |
| **DOPE score** | -52937.51172 | -52808.47656 | -53014.12500 | -52638.96484 | -52499.44141 |
| ***L. major* Trypanothione reductase (*Lm*TR)** | | | | | |
|  | ***Lm*TR I** | ***Lm*TR II** | ***Lm*TR III** | ***Lm*TR IV** | ***Lm*TR V** |
| **DOPE score** | -53008.44531 | -52964.11719 | -52707.29297 | -52769.12109 | -52834.90625 |
| ***L. donovani* Glutamate cysteine ligase (*Ld*GCL)** | | | | | |
|  | ***Ld*GCL I** | ***Ld*GCL II** | ***Ld*GCL III** | ***Ld*GCL IV** | ***Ld*GCL V** |
| **DOPE score** | -71943.04688 | -72709.16406 | -72252.65625 | -70272.20313 | -73083.05469 |
| ***L. major*** **Glutamate cysteine ligase (*Lm*GCL)** | | | | | |
|  | ***Lm*GCL I** | ***Lm*GCL II** | ***Lm*GCL III** | ***Lm*GCL IV** | ***Lm*GCL V** |
| **DOPE score** | -72157.15625 | -71900.67969 | -72528.82813 | -72161.99219 | -73102.12500 |

Table S2: Predicted binding sites for the 3 modelled receptors using CASTp. The sizes of the volumes, areas and residues lining the pockets are provided.

| **RECEPTOR** | **POCKET No.** | **Volume** | **Pocket**  **Area** | **RESIDUES** |
| --- | --- | --- | --- | --- |
| *Ld*TR III | 1 | 6104.8 | 3148.9 | Leu10, Gly11, Ala12, Gly13, Ser14, Gly15, Gly16, Val34, Asp35, Val36, Leu44, Phe45, Ala46, Ala47, Gly50, Thr51, Cys52, Val55, Gly56, Cys57, Lys60, Lys61, Gly125, Phe126, Gly127, Glu141, Ala159, Thr160, Gly161, Ser162, Trp163, Pro164, Thr165, Arg166, Leu167, Thr177, Ser178, Asn179, Phe182, Val194, Gly195, Gly196, Gly197, Tyr198, Ile199, Ala200, Glu202, Phe203, Tyr221, Arg222, Leu227, Arg228, Gly229, Phe230, Leu253, Asn254, Pro255, Leu283, Ala284, Ile285, Gly286. Arg287, Val288, Pro289, Arg290, Gln292, Ala293, Leu294, Lys305, Asn306, Gln310, Val311, Ile325, Gly326, Asp327, Thr329, Asn330, Arg331, Val332, Met333, Leu334, Thr335, Pro336, Val337, Ala338, Asn340, Glu341, Thr357, Asp358, His359, Thr360, Lys361, Val362, Ala363, Cys364, Ala365, Val366, Phe367, Thr374, Cys375, Gly376, Met377, Thr378, Glu380, Glu381, Lys384, His428, Gln439, Gly442, Ile443, Lys446. |
|  | 2 | 439.4 | 222.6 | Phe174, Ile176, Tyr183, Leu184, Glu185, Asp186, Pro188, Lys260, Asp263, Gly264, Ser265, Asn266, Asp279, Gln280. |
|  | 3 | 378.8 | 228.4 | Ile437, Ser440, Val441, Cys444, Ala449, Asp453, Phe454, Thr457, Ile458, Gly459, Val460, Ala465, Glu466, Leu468, Cys469. |
|  | 4 | 49.123 | 101.965 | Lys61, Val64, Thr65, Gln68, Asp71, Leu72, Glu75, Phe367, Ser368, Ile369, Pro370, Pro371, His401, Lys409, Asp432, Pro435. |
| *Lm*TR I | 1 | 3604.3 | 2113.3 | Leu10, Gly11, Ala12, Gly13, Ser14, Gly15, Gly16, Val34, Asp35, Val36, Phe44, Ala46, Ala47, Gly50, Thr51, Cys52, Val55, Gly56, Cys57, Lys60, Gly125, Phe126, Gly127, Glu141, Ala159, Thr160, Gly161, Ser162, Trp163, Ser178, Phe182, Gly195, Gly196, Gly197, Tyr198, Ile199, Glu202, Phe203, Tyr221, Arg222, Leu227, Arg228, Gly229, Phe230, Asp231, Ala284, Ile285, Gly286, Arg287, Arg290, Thr293, Leu294, Ile325, Gly326, Asp327, Asn330, Arg331, Val332, Met333, Leu334, Thr335, Pro336, Val337, Ala338, Asn340, Arg355, Ala356, Thr357, Asp358, His359, Thr360, Lys361, Val362, Ala363, Cys364, Ala365, Val366, Phe367, Thr374, Cys375, Gly376, Met377, Thr378, Glu381, His428, Gln439, Gly442, Ile443, Lys446. |
|  | 2 | 389.6 | 218.9 | Ile437, Ser440, Val441, Cys444, Ala449, Asp453, Phe454, Thr457, Ile458, Gly459, Val460, Ala465, Glu466, Leu468, Cys469. |
|  | 3 | 287.5 | 125.7 | Leu184, Glu185, Asp186, Ala187, Pro188, Lys189, Asp263, Gly264, Ser265, Asp279, Asp279, Gln280 |
|  | 4 | 437.3 | 307.9 | Gly66, Ala67, Tyr69, Met70, Ile73, Arg74, Arg85, Glu86, Leu88, Cys89, Pro90, Asn208, Gly209, Tyr210, Lys211, Pro212, Arg213. |
| *Ld*GCL V | 1 | 5171.6 | 2193.8 | Arg190, Cys195, Val196, Leu197, Val198, Pro199, Leu200, Tyr201, Met202, Ser204, Arg205, Thr206, Met207, Gln208, Asp218, Thr220, His222, Asn223, Asp225, Ile226, Phe227, Tyr228, Ser229, Met230, Asn233, Gly234, Arg235, Asn236, Met237, Thr238, Asp239, Glu240, Tyr242, Ala243, Glu244, His310, Pro311, Cys312, Gln363, Asp369, Thr370, Val372, Arg373, Trp374, Leu375, Thr376, Ala378, Gly379, Asp382, Arg384, Ile392, Leu393, Lys394, Ser395, Asp398, Ser399, Ile400, Ser401, Ser417, Gln418, Ile419, Ala420, Ile534, Ser535, Val537, Asp538, Glu539, Met541, Gly542, Arg543, His545, Arg546, Ile547, Asn548, Cys550, Gly551, Glu552. |
|  | 2 | 3083.2 | 1978.4 | Met1, Gly2, Leu3, Leu4, Thr5, Thr6, Phe33, Val36, Pro47, Leu49, Trp50, Gly51, Glu52, Glu53, Glu55, Ala73, Met76, Asn77, Val88, Asn90, Pro91, Glu92, Tyr93, Met97, Glu99, Ser100, Thr101, Pro102, Asp103, His104, Pro105, Thr175, His176, Pro177, Arg178, Phe179, Leu182, Met318, Gly321, Met322, Asn324, Ser325, Ala326, Gln328, Val329, Thr330, Met331, Gln332, Arg373, Trp374, Ile377, Tyr397, Val452, Met453, Tyr454, Met457, Thr466, Glu467, Phe469, Asp470, Asn471, Gln473, Ser474, Trp477, Gln478, Arg481, Lys483, Pro484, Pro485, Pro486, Asn489, Asp490, Ile491, Arg494, Glu496, Arg498. |
|  | 3 | 1849.0 | 1466.6 | Phe48, Trp50, Leu108, Ser109, Val110, Glu111, Ser112, Asp114, Gln345, Leu346, Ile348, Leu349, Ala352, Phe353, Val514, Phe515, Pro517, Leu518, Leu519, Lys521, Ala522, Ile523, Tyr526, Pro528, Phe530, Met556, Arg557, Asp559, Ile560, Phe561, Ile580, Phe581, Asn582, Gly583, Lys584, Glu585, Gly586, Gly587, Phe588, Tyr589, Gly590, Leu591, Ile592, Pro593, Leu594, Val595, Arg597, Tyr598, Leu599, Asp600, Asp601, Glu602, Gly603, Ser606, Pro607, Leu608, Val609, Asn610, Tyr612, Leu613. |
|  | 4 | 691.6 | 421.9 | Gly149, Asn150, Phe151, Val152, Cys154, Ser155, Asp156, Lys157, Ser158, Ser159, Gln163, Ser164, Leu165, Phe166, Val167, Pro168, Ser257, Asp259, Pro260, Arg261, Glu262, Leu273, Phe274. |
| *Lm*GCL V | 1 | 1226.690 | 994.906 | Met1, Gly2, Leu3, Leu4, Thr5, Gln32, Phe33, Val36, Phe49, Trp50, Gly51, Glu52, Glu53, Glu55, Val88, Asn90, Pro91, Glu92, Tyr93, Met97, Glu99, Thr101, Pro102, Asp103, His104, Pro105, Arg178, Phe179, Leu182, Met318, Gly321, Met322, Asn324, Ser325, Ala326, Gln328, Val329, Thr330, Met331, Gln332, Arg373, Trp374, Ile377, Tyr397, Val452, Met453, Tyr454, Met457, Thr466, Glu467, Phe469, Asp470, Asn471, Gln473, Ser474, Trp477, Gln478, Arg481, Lys483, Pro484, Pro485, Pro486, Ile491, Arg494, Glu496, Arg498. |
|  | 2 | 600.141 | 721.973 | Thr70, Ala73, Ser95, Gly147, Gln148, Gly149, Asn150, Phe151, Val152, Cys154, Ser155, Asp156, Lys157, Ser158, Ser159, Gln163, Ser164, Leu165, Phe166, Val167, Pro168, Ala170, Cys171, Ile172, Asn173, Gln174, Thr175, Asp239, Leu241, Leu252, Val253, Pro254, Ser255, Ser256, Ser257, Asp259, Pro260, Arg261, Glu262, Asp263, Tyr264, Pro265, Leu273, Lys364, Gly365, Leu366, Arg646. |
|  | 3 | 147.435 | 108.037 | Gln12, Ser158, Pro160, Tyr161, Gln163, Ala184, Arg187, Leu188, Tyr264, Leu270, Lys271, Gln272, Leu273, Phe274. |
|  | 4 | 118.392 | 316.376 | Phe48, Trp50, Leu108, Ser109, Val110, Glu111, Asp114, Val514, Pro517, Leu518, Lys521, Asn582, Gly583, Lys584, Tyr589, Gly590, Leu591, Ile592, Leu594, Val595, Tyr598, Leu599, Asp600, Asp601, Glu602, Gly603, Val609, Asn610, Leu613. |
